# Maternal Wnt11b regulates cortical rotation during *Xenopus* axis formation: analysis of maternal-effect *wnt11b* mutants

**DOI:** 10.1242/dev.200552

**Published:** 2022-09-09

**Authors:** Douglas W. Houston, Karen L. Elliott, Kelsey Coppenrath, Marcin Wlizla, Marko E. Horb

**Affiliations:** 1 Department of Biology, The University of Iowa, 257 BB, Iowa City, IA 52242-1324, USA; 2 National Xenopus Resource and Eugene Bell Center for Regenerative Biology and Tissue Engineering, Marine Biological Laboratory, Woods Hole, MA 02543, USA

**Keywords:** *Xenopus*, Dorsal axis, Wnt11, Wnt11b, Microtubules, Cortical rotation, CRISPR/Cas9

## Abstract

Asymmetric signalling centres in the early embryo are essential for axis formation in vertebrates. These regions (e.g. amphibian dorsal morula, mammalian anterior visceral endoderm) require stabilised nuclear β-catenin, but the role of localised Wnt ligand signalling activity in their establishment remains unclear. In *Xenopus*, dorsal β-catenin is initiated by vegetal microtubule-mediated symmetry breaking in the fertilised egg, known as ‘cortical rotation’. Localised *wnt11b* mRNA and ligand-independent activators of β-catenin have been implicated in dorsal β-catenin activation, but the extent to which each contributes to axis formation in this paradigm remains unclear. Here, we describe a CRISPR-mediated maternal-effect mutation in *Xenopus laevis wnt11b.L*. We find that *wnt11b* is maternally required for robust dorsal axis formation and for timely gastrulation, and zygotically for left-right asymmetry. Importantly, we show that vegetal microtubule assembly and cortical rotation are reduced in *wnt11b* mutant eggs. In addition, we show that activated Wnt coreceptor Lrp6 and Dishevelled lack behaviour consistent with roles in early β-catenin stabilisation, and that neither is regulated by Wnt11b. This work thus implicates Wnt11b in the distribution of putative dorsal determinants rather than in comprising the determinants themselves.

This article has an associated ‘The people behind the papers’ interview.

## Abstract (precis)

**Summary:** Maternal Wnt11b regulates microtubule dynamics during cortical rotation.

## INTRODUCTION

The formation of a single dorsal midline axis of bilateral symmetry in the embryo is a central event in the early development of vertebrate animals. In amphibians, this process depends on symmetry-breaking in the fertilised egg, which occurs through a process of microtubule-mediated ‘cortical rotation’ of the egg cortex, resulting in corticocytoplasmic translocation of putative dorsal axial determinants (Gerhart, 2004; Houston, 2012, 2017; Weaver and Kimelman, 2004). A major outcome of cortical rotation is the stabilisation and nuclear localisation of β-catenin (also known as Ctnnb1) during the 16- to 32-cell stages (Kao and Elinson, 1988; Yang et al., 2002). This enrichment of β-catenin sets up chromatin states and later gene regulatory interactions (Blythe et al., 2010) that lead to the specification of cells comprising the Spemann organiser (reviewed by Houston, 2017). The organiser ultimately elaborates axial patterning through the formation of extracellular gradients of BMP (and other) growth factor antagonists.

At least two non-mutually exclusive views of dorsal signalling subsequent to cortical rotation have been considered, including the intracellular localisation of cytoplasmic activators of Wnt signalling in the absence of Wnt ligand and the dorsal enrichment of vegetally localised Wnt ligand (*wnt11b*) mRNA. The (first) intracellular localisation model is supported by various studies, including: cytoplasmic ablation/transplantation experiments involving egg cytoplasm (Darras et al., 1997; Kageura, 1997; Marikawa and Elinson, 1999; Marikawa et al., 1997), the visualisation of translocating particles containing exogenous Dishevelled (Dvl) and/or Frat1/GBP fusion proteins (Miller et al., 1999; Weaver et al., 2003) and the failure of overexpressed extracellular Wnt antagonists to inhibit endogenous β-catenin activity or axis determination (Hoppler et al., 1996; Leyns et al., 1997; Wang et al., 1997).

Also, recent work in zebrafish and frogs has shown that deficiency in maternal *huluwa* (*hwa*; Yan et al., 2018), which encodes a localised RNA in both species, results in ventralisation. These data also included dominant-negative results suggesting independence from secreted Wnt signalling, supporting previous results in frog embryos (Hoppler et al., 1996; Leyns et al., 1997; Wang et al., 1997). Hwa accumulates dorsally and likely promotes the dorsal degradation of Axin1, a key scaffold protein that facilitates β-catenin degradation (Yan et al., 2018). More broadly, similar evidence is accumulating in mice that the anterior visceral endoderm (AVE), the requisite axis-regulating region, can form in the absence of secreted Wnt ligand signalling but requires nuclear β-catenin activity (reviewed by Houston, 2017).

The (second) extracellular signalling model was suggested by experiments showing that depletion of maternal *wnt11b* mRNA using antisense oligonucleotides (oligos) results in axial defects, with corresponding loss of early β-catenin target gene expression (Tao et al., 2005). These studies also indicated that Wnt11b is required extracellularly, in a complex with Exostosin glycosyltransferase 1 (Ext1) and Tdgf1 (Cripto/FRL-1) homologues, and possibly with other Wnt ligands (Cha et al., 2008; Tao et al., 2005), implying an extracellular signalling event. TALEN-mediated mutagenesis in zebrafish has ruled out a maternal role for vegetally localised *wnt8a* RNA in the early development of this species (Hino et al., 2018). The maternal roles of other Wnts have yet to be tested in zebrafish.

These studies on maternal Wnt signalling need to be reconciled with more general data on Wnt/β-catenin signalling showing the importance of activated Wnt receptor-coreceptor complexes (Lrp6 ‘signalosomes’) in association with Dvl (Bilić et al., 2007). A hybrid model of maternal β-catenin regulation has been proposed, wherein cortical rotation might enrich active phospho-Lrp6 signalosomes on the dorsal side (Dobrowolski and De Robertis, 2012). However, recent data suggest that endocytosis may not be required for Wnt/β-catenin activation (Rim et al., 2020). And, the extent to which phospho-Lrp6 signalosomes become dorsally enriched is unknown.

To re-evaluate the role of maternal Wnt11b signalling in axis formation, we used targeted genome editing to generate *Xenopus laevis* deficient in *wnt11b*, obtaining embryos lacking Wnt11b function maternally, zygotically or both. Our data show that maternal Wnt11b signalling is required for proper initiation of dorsal gene expression, axis formation and gastrulation morphogenesis, but that axial tissues eventually form in many cases. In addition, consistent with previous reports, we find that loss of Wnt11b activity is associated with left-right asymmetry defects. Regulation of Lrp6 phosphorylation and Dvl puncta were unchanged in *wnt11b* mutant oocytes and eggs. Furthermore, and in contrast to the existing models for Wnt11b in β-catenin activation, live imaging experiments show that Wnt11b function is required for normal vegetal microtubule dynamics and organisation, implicating Wnt11b in the initiation or maintenance of, rather than (or in addition to) the outcome of, cortical rotation.

## RESULTS

### Mutagenesis of *Xenopus laevis wnt11b*

CRISPR/Cas9 mutagenesis was used to create insertion-deletions (indels) in the *wnt11b.L* locus of the inbred *X. laevis* J-strain (Session et al., 2016; Tochinai and Katagiri, 1975). These mutants were generated and raised at the National *Xenopus* Resource (NXR; Pearl et al., 2012). We designed guide RNAs targeting the first two exons of *wnt11b.L* on chromosome 8L (hereafter *wnt11b*) (Fig. S1A; Table S1). These guides lack homology to the paralogous *wnt11.L/S* genes (*wnt11-r*), which are located on chromosomes 2L/2S and are not expressed maternally, but have zygotic expression patterns similar to *wnt11b* (Garriock et al., 2005) (Fig. S1B). The homologue (alloallele) for *wnt11b.S*, which would be present on chromosome 8S, is absent, a likely consequence of widespread gene loss on the S subgenome chromosomes (Session et al., 2016). *X. laevis* also lacks a duplicated *wnt11b* gene found in *Xenopus tropicalis* (*xetro.H00536*; Dichmann et al., 2015)*.* Thus, *X. laevis* is functionally diploid for the *wnt11b* gene, and Wnt11b represents the sole contribution to maternal Wnt11 protein family function.

**Fig. 1.**
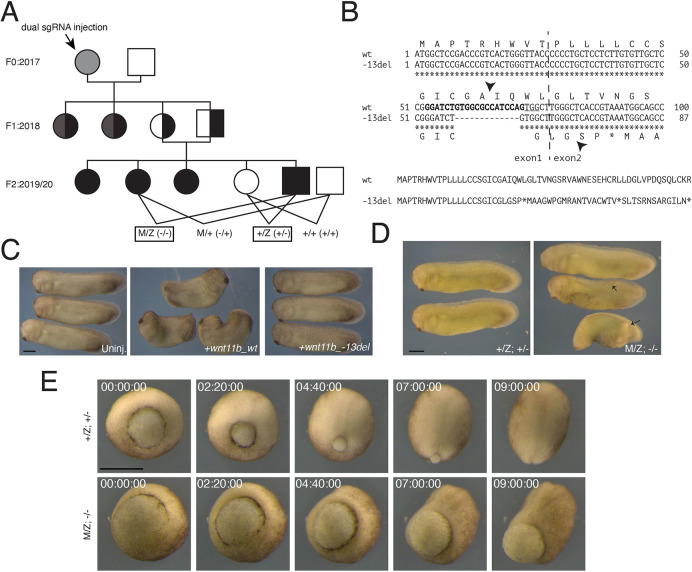
**Mutagenesis of *wnt11b.L* in *Xenopus laevis*.** (A) Genealogy of *Xla.wnt11b^emNXR^* maternal-effect mutants. Females are represented by circles, males are represented by squares. The symbol in light grey indicates F0 mosaic; black indicates mutant at sgT1 site; dark grey indicates mutant at sgT2 site; full shading indicates homozygosity; half shading indicates heterozygosity. (B) Alignments of wild-type (wt) and mutant (–13del) nucleotide and predicted protein sequences. The CRISPR sgRNA target is in bold; protospacer adjacent motif is underlined. The vertical dashed line represents the exon 1-exon 2 boundary. Arrowheads indicate predicted signal peptide cleavage sites. (C) Embryos injected with *wnt11b* wild-type mRNA (*wnt11b_wt*; middle), *wnt11b* mutant mRNA (*wnt11b_–13del*; right) or uninjected (Uninj.; left). (D) Images of heterozygotes (+/Z; left) and maternal-zygotic *wnt11b* mutants (M/Z; right). Arrows indicate ectopic tailbuds. (E) Representative frames from time-lapse movies of a heterozygous control gastrula (+/Z; top) and a maternal-zygotic *wnt11b* gastrula (M/Z; bottom). Time stamps indicate time from the beginning of filming (h:min:s). Scale bars: 500 µm.

Guide RNAs against *wnt11b* were complexed with Cas9 protein and injected into one-cell embryos obtained from matings/*in vitro* fertilisations of wild-type J-strain *X. laevis* to begin generating a mutant pedigree (Fig. 1A). A set of resulting embryos were grown to sexual maturity and females were tested for germline transmission of indels. An F0 female was identified that transmitted a 13 bp deletion (−13del; allele designation *Xla.wnt11b^emNXR^*), located near the 3′ end of exon 1 (sgT1 guide RNA site). This deletion is predicted to generate a frameshift mutation resulting in a premature stop codon just after the Wnt11b signal peptide sequence cleavage site (Fig. 1B). Outcrossing of this ‘founder’ female to a wild-type J strain male resulted in heterozygous F1 progeny that were then intercrossed. Three sexually mature F2 females homozygous for the 13 bp deletion were initially identified, in addition to several homozygous mutant males (Fig. 1A). These F2 generation *Xla.wnt11b^emNXR/emNXR^* (*wnt11b*^−/−^ hereafter) frogs all developed normally (outwardly), indicating that zygotic *wnt11b* is not uniquely required for developmental viability.

To assess the functionality (or lack thereof) of the *−13del/emNXR* allele, we amplified and cloned the *wnt11b.L* coding region cDNAs from wild-type and mutant oocyte RNA by RT-PCR. Sequencing confirmed that the 13 bp deletion created the predicted frameshift mutation, resulting in premature stop codons in the expressed RNA, and that it did not disrupt normal exon 1-exon 2 splicing (Fig. 1B). In addition, we synthesised transcripts from these cDNAs *in vitro* and assessed their activity through overexpression in wild-type *Xenopus* embryos. In all cases (*n*≥30 each; two experiments), wild-type *wnt11b* induced axis shortening, consistent with known phenotypic effects of *wnt11b* injection (Fig. 1C; Du et al., 1995). By contrast, overexpression of mutant *wnt11b-13del* transcripts had no effect on development and morphogenesis (Fig. 1C), a result further in line with this mutant allele lacking activity.

### *wnt11b* is maternally required for normal progression through gastrulation and axial morphogenesis

Homozygous *wnt11b^−/−^* F2 females produced fertile eggs, and homozygous mutant F2 males were similarly fertile, indicating that zygotic Wnt11b function is not required for overall germline development. To assess the role of maternal Wnt11b in development, we fertilised eggs from homozygous mutant females using sperm from either wild-type or mutant males, alongside eggs from wild-type females. Because we wished to unambiguously describe the strictly maternal effects of *wnt11b* loss-of-function, we designated the origin of the mutant allele in these crosses using a capital ‘M’ or ‘Z’ for whether the mutant allele was maternally/egg derived (‘M’) or zygotically/sperm derived (‘Z’). Thus maternal-zygotic mutants are referred to using ‘M/Z’ in this work, whereas heterozygous embryos are indicated as ‘M/+’ or ‘+/Z’, corresponding to mutant or wild-type (+) eggs fertilised with wild-type or mutant sperm, respectively. For simplicity, most experiments focused on comparing M/Z (MZ*wnt11b^−/−^*) embryos with +/Z (+Z*wnt11b^+/−^*) embryos.

The crosses described above (Fig. 1A) resulted in embryos that developed normally until gastrulation, without visible changes in early cell cycles. In embryos derived from mutant females (M/Z and M/+), dorsal lip formation was initiated on time in roughly half the cases (Fig. S2A-C), but the progression through gastrulation was consistently delayed with complete penetrance, regardless of paternal genotype (see below). Maternal mutant embryos subsequently developed a range of defects in embryogenesis (variable expressivity), ranging from severe axial truncation/ventralisation, with or without open blastopores and neural tubes (spina bifida-like) and small ectopic tailbuds, to largely normal development (Fig. 1D; Table 1). We characterised the delays in gastrulation using time-lapse imaging of heterozygous (+/Z) and maternal/zygotic homozygous (M/Z) embryos. For these experiments, embryos were fertilised at the same time and imaged in the same dish (Fig. 1E; Movie 1). Movies were started when the control +/Z embryos were at the mid-gastrula stage (stage 10.5; Nieuwkoop and Faber, 1956), the time when the majority of M/Z embryos were unequivocally delayed (Fig. 1E, time 00 h:00 min:00 s). M/Z embryos did not initiate ventral blastopore closure until the equivalent of stage 12 and most did not fully close the blastopore during the 9 h of imaging. Neural plate formation and morphogenesis commenced roughly on schedule in these abnormal embryos, suggesting a primary defect in gastrulation and not in dorsal signalling per se (Fig. 1E, time 07 h:00 min:00 s). Similar results were seen using antisense morpholino oligos against all wnt11 family transcripts (Van Itallie et al., 2022 preprint), suggesting that normal expression of either maternal (of which Wnt11b is the sole contributor) or pan-zygotic Wnt11 proteins is required for timely and complete gastrulation. Because gastrulation involves coordinated regulation of the cytoskeleton, we examined cytoskeletal organisation in +/Z and M/Z embryos. Gastrula embryos were stained for filamentous actin, tubulin and cytokeratin. No major differences were seen in staining for these cytoskeletal systems, although there appeared to be excess cytokeratin filaments in the mutants, which could indicate slower turnover, possibly affecting the rate of morphogenesis (Fig. S2D-I).

**Table 1.**
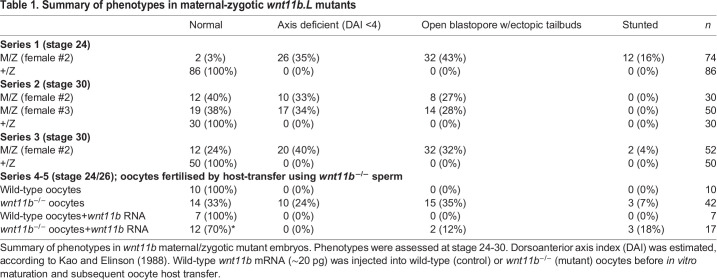
Summary of phenotypes in maternal-zygotic *wnt11b.L* mutants

The formation of normal dorsal axial structures was first verified by assessing antigen tissue markers at the tailbud stage. Immunostaining against notochord and somite antigens using monoclonal antibodies (mAbs) Tor70 and 12/101 (Bolce et al., 1992; Kintner and Brockes, 1984; Kushner, 1984) showed reduced and disorganised expression in the population of mutant embryos that formed dorsal axes (Fig. S3). Interestingly, immunostaining against NCAM (mAb 6F11; Lamb et al., 1993) revealed ectopic neural differentiation both in ectopic tailbud structures and in epidermal regions throughout the embryo (Fig. S3). Embryos lacking visible axes lacked staining for these antigens (not shown). Thus, whereas a subset of embryos maternally deficient in *wnt11b* lack dorsal axes, many are able to form dorsal structures and even ectopic neural tissues.

### Wnt11 family transcript expression is normal in *wnt11b* mutant oocytes and embryos

**Fig. 2.**
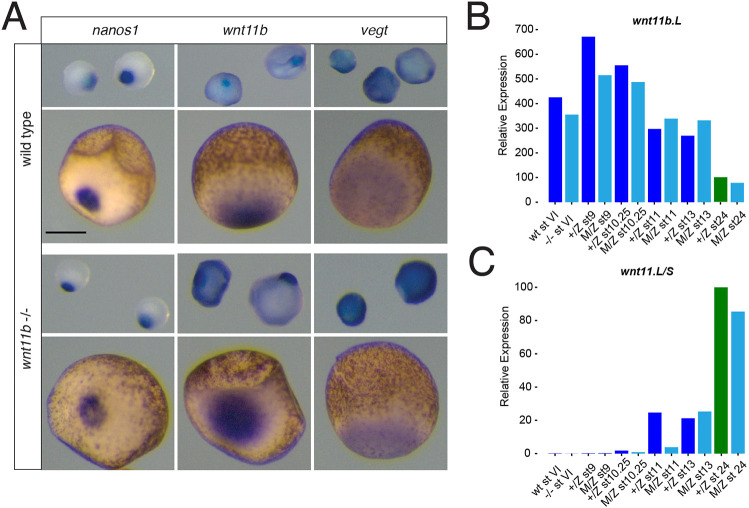
**Normal expression of *wnt11b* in mutants.** (A) *In situ* hybridisation of *nanos1*, *wnt11b* and *vegt* expression in oocytes from wild-type and homozygous mutant females (stages I-II upper row in each set; stages IV lower rows; vegetal views). (B,C) Real-time RT-PCR analysis of *wnt11b* (B) and *wnt11* (C) in wild-type and mutant oocytes (stage VI) and in heterozygotes and maternal-zygotic mutant embryos. Staging is by Nieuwkoop and Faber (1956). Green bars are the stage (st24) used for relative expression; mutant samples are in cyan. Scale bar: 250 µm.

We next sought to determine the extent to which the early stop codon in the *emNXR* mutant allele of *wnt11b* may have indirectly affected activities unrelated to Wnt11b protein function, resulting from either the degradation or delocalisation of *wnt11b* transcripts during oogenesis. We also examined whether compensatory expression of *wnt11.L/S* genes (on chromosome 2) might have occurred. We isolated oocytes and embryos from *wnt11b* homozygous mutant females and from wild-type siblings and analysed the expression of RNAs localised to the vegetal cortex. RNAs representative of the three main classes of localised RNAs were examined: germ plasm, late pathway and intermediate RNAs, exemplified by *nanos1*, *vegt* and *wnt11b* itself (Houston, 2013). All three RNAs were expressed similarly when comparing wild-type and mutant oocytes (Fig. 2A), indicating that general oocyte polarisation and RNA localisation occur normally in *wnt11b^−/−^* mutant animals.

We also assessed *wnt11b* and *wnt11* RNA expression in oocytes and embryos by RT-PCR. Transcripts for *wnt11b* were expressed equivalently in wild-type and *wnt11b^−/−^* mutant oocytes and embryos throughout development (Fig. 2B), suggesting a lack of nonsense-mediated decay maternally. *wnt11.L/S* paralogues were not ectopically expressed maternally in *wnt11b^−/−^* mutant oocytes or during later stages (Fig. 2C), although their expression was delayed until around stage 11 (mid-gastrulation), likely as part of the general delay in gastrulation in these embryos (see below).

These data suggest that the variable defects seen in *wnt11b* maternal mutants are the result of loss of maternal Wnt11b protein function, and that variable up- or downregulation of the paralogous *wnt11.L/S* genes or disruption of RNA localisation does not generally occur and does not appear to contribute to the *wnt11b*-deficient phenotypes.

### *wnt11b* is required zygotically for left-right asymmetry

In the course of these initial studies on the role of maternal *wnt11b*, we noted that a homozygous *wnt11b^−/−^* F2 male used as a testis donor had reversed organ situs. Because *wnt11b* had previously been implicated in laterality signalling using morpholino-based knockdown (i.e. loss of left-sided *pitx2c* expression; Walentek et al., 2013), we first performed heterozygous crosses to assess laterality genetically. We scored resulting embryos at the swimming tadpole stage, when organ laterality is easily discernible (Yost, 1992). In a pilot experiment (47 total tadpoles), we identified three tadpoles that exhibited reversed heart orientation (heterotaxy or situs inversus; abnormal gut coiling and/or heart orientation). Genotyping revealed that all three heterotaxic tadpoles were homozygous for *wnt11b^−/−^*, whereas the remaining eight homozygous mutant tadpoles, and the heterozygotes and wild-type tadpoles, had normal situs (situs solitus; Table 2), a frequency of ∼25% laterality defects in homozygous F2 mutants.

**Table 2.**
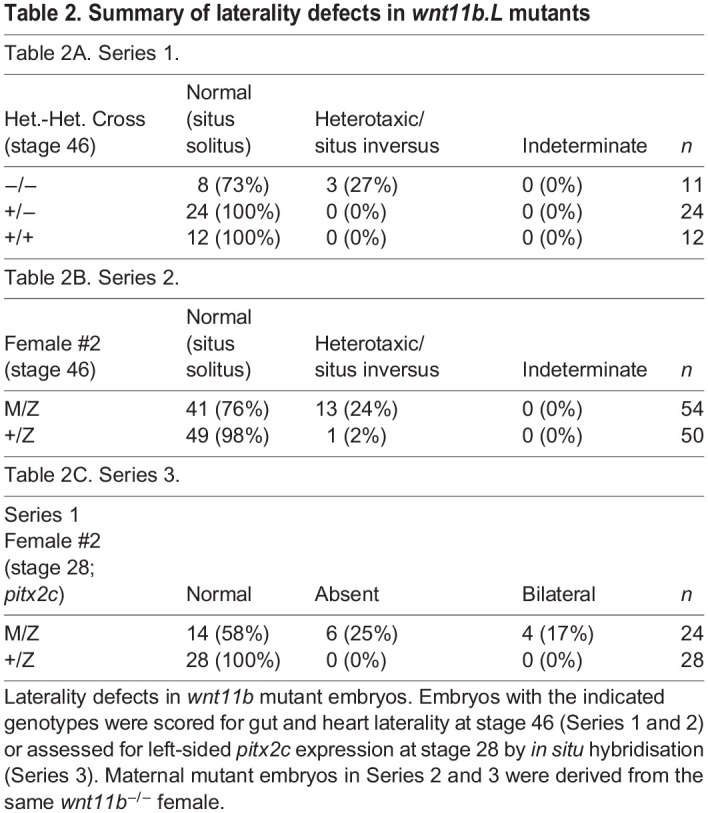
Summary of laterality defects in *wnt11b.L* mutants

Table 2B. Series 2.				
Female #2 (stage 46)	Normal (situs solitus)	Heterotaxic/situs inversus	Indeterminate	*n*
M/Z	41 (76%)	13 (24%)	0 (0%)	54
+/Z	49 (98%)	1 (2%)	0 (0%)	50

Table 2C. Series 3.				
Series 1				
Female #2 (stage 28; *pitx2c*)	Normal	Absent	Bilateral	*n*
M/Z	14 (58%)	6 (25%)	4 (17%)	24
+/Z	28 (100%)	0 (0%)	0 (0%)	28

**Fig. 3.**
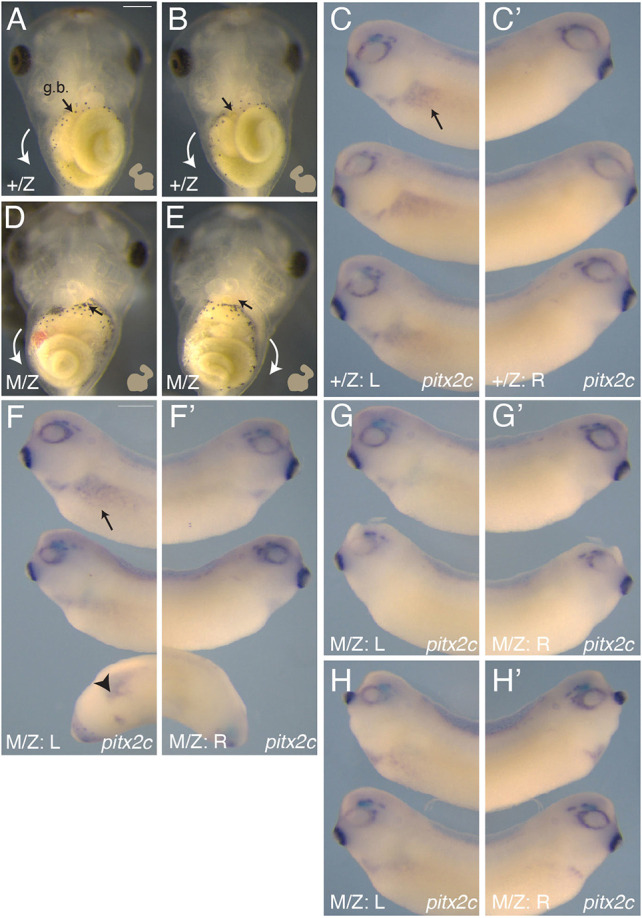
**Laterality defects in *wnt11b* mutant embryos.** (A,B) Ventral views of stage 46 control heterozygotes (+/Z), showing normal lateral asymmetry. The heart shape is diagrammed (lower right), showing the ventricle positioned on the left-side and the outflow tract oriented to the right. The intestines coil anticlockwise (white arrows); g.b., gallbladder (arrow). (C) Left-sided *pitx2c* expression at stage 30 in heterozygous controls (arrow). (C′) Absence of *pitx2c* expression on the right side of control embryos. (D,E) Ventral views of homozygous maternal-zygotic mutants (M/Z) at stage 46, showing examples of heterotaxy and situs inversus, respectively. The heart orientation is reversed in both examples, and the gut coils clockwise in E. Arrows are as above. (F-H′) *pitx2c* expression in M/Z mutants; (F,F′) examples of embryos with normal *pitx2c* expression patterns (left-sided expression only); (G,G′) examples lacking expression on left and right sides, respectively; (H,H′) examples of bilateral *pitx2c* expression in a subset of M/Z mutant embryos. Arrowhead in F indicates example of normal *pitx2c* expression in an axially truncated embryo. Scale bars: 500 µm.

Subsequent observations of gut and heart morphogenesis in surviving F3 MZ*wnt11b^−/−^* mutant swimming tadpoles (with outwardly normal development) showed a similar frequency of heterotaxy (24-25%; *n*≥50; Table 2; Fig. 3A,B,D,E). Examination of *pitx2c* expression at the tailbud stage showed normal expression in the left lateral plate mesoderm of heterozygous embryos (+/Z; Fig. 3C,C′) and in about half the maternal-zygotic mutants (M/Z; Fig. 3F,F′). Left-sided *pitx2c* could be seen in some axis-deficient embryos, suggesting left-right defects could be independent from axial induction defects (Fig. 3F). The remaining maternal-zygotic mutant embryos lacked or were severely reduced in left-sided *pitx2c* expression (Fig. 3G,G′), or exhibited bilateral *pitx2c*, with weak expression on the right side (Fig. 3H,H′; Table 2). These frequencies are comparable with the frequency seen in the null embryos from heterozygous matings (above), and in published studies on *wnt11b* morpholino-injected embryos (in which zygotic, but not maternal, Wnt11b function is affected; Walentek et al., 2013). These congruences suggest that the function of Wnt11b in left-right signalling is mainly performed by zygotically expressed *wnt11b*. We did not pursue this zygotic aspect of Wnt11b function further.

### Maternal Wnt11b is required for normal early dorsal-ventral gene expression

**Fig. 4.**
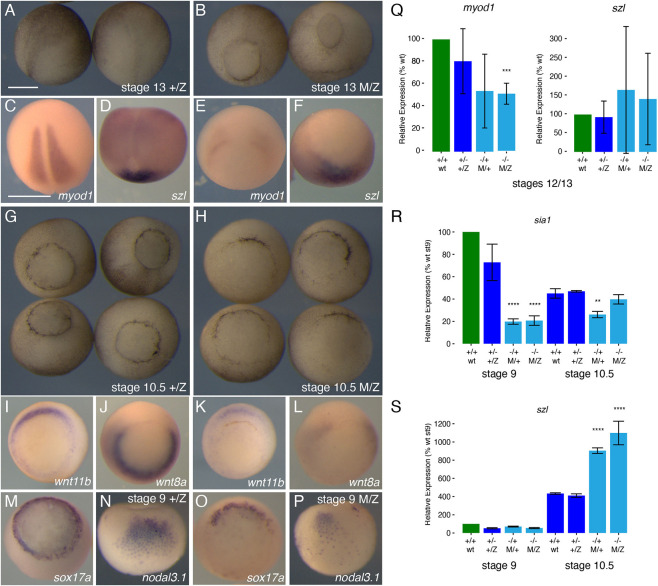
**Delayed and reduced mesendodermal gene expression in maternal *wnt11b* mutants*.*** (A,C,D,G,I,J,M,N) Phenotypes (A,G) and *in situ* hybridisation of mesendodermal markers (C, *myod1*; D, *szl*; I, *wnt11b*; J, *wnt8a*; M, *sox17a*; N, *nodal3.1*) in early neurula (A,C,D) and mid/early gastrulae (G,I,J,M,N) in controls (+/Z; left-hand panels). (B,E,F,H,K,L,O,P) Phenotypes (B,H) and *in situ* hybridisation of mesendodermal markers (E, *myod1*; F, *szl*; K, *wnt11b*; L, *wnt8a*; O, *sox17a*; P, *nodal3.1*) in early neurula stage (B,E,F) and mid/early gastrulae (H,K,L,O,P) in maternal mutants (M/Z; right-hand panels). Dorsal, posterior views (A-F); vegetal views (G-P, dorsal towards top). (Q-S) Real-time RT-PCR of *myod1* and *szl* at stages 12/13 (Q), and *sia1* (R) and *szl* (S) at stages 9 and 10.5. Green indicates the samples used for normalisation; maternal mutants are coloured cyan. Error bars represent the s.d. of two biological replicates (pools of three embryos); unpaired two-tailed *t*-test; **P<0.01, ***P<0.001, ****P<0.0001. Scale bars: 500 µm (in A for A,B,G,H; in C for C-F,I-P).

To characterise the maternal effects of *wnt11b* loss-of-function, we analysed gene expression in embryos derived from *wnt11b^−/−^* F2 females at the gastrula and neurula stages, when visible phenotypes first became apparent. Consistent with visible delays in gastrulation, the expression of mesoderm and endoderm markers was reduced and delayed during gastrulation in maternally *wnt11b*-deficient embryos (Fig. 4). Notably, mesodermal genes *myod1* (stages 12-13), *wnt11b* itself and *wnt8a* (stage 10.5) were expressed in patterns consistent with delayed blastopore formation in maternal mutant embryos (Fig. 4A-L,Q). This delay was particularly evident in delayed ventral endoderm expression of *sox17a* (Fig. 4M,O), whereas a general ventral fate marker, *sizzled* (*szl*), was expressed in its normal spatial pattern (Fig. 4D,F,Q), although at elevated overall levels. The expression of dorsal and Wnt/β-catenin target genes was also reduced in maternal *wnt11b* mutant embryos (MZ*wnt11b^−/−^* and M+*wnt11b^−/+^*). *In situ* hybridisation analyses of dorsal *nodal3.1* expression showed either reduced or absent expression in all cases, with ∼50% lacking visible signal (Fig. 4N,P; Fig. S4). Similarly, in RT-PCR analyses, levels of the organiser marker *siamois homeodomain 1* (*sia1*; Fig. 4R) and *nodal3.1* (Fig. S4) were reduced to ≤20% of control levels (on average) in embryos derived from *wnt11b* mutant eggs (M/Z and M/+). Conversely, expression of the ventral marker *szl* was expressed at normal (low) levels at stage 9, but was elevated in maternal *wnt11b* mutant embryos at stage 10.5 (Fig. 4S).

Preliminary RNA-sequencing analysis showed that other mesendodermal genes (e.g. *mixer*, *foxc2*) and ectodermal/prospective neuroectoderm markers (e.g. *lhx5*, *sox2*) were either unaffected or slightly changed in mutants (up or down; Fig. S5A,B). Levels of *wnt5a.L/S* were slightly elevated in MZ*wnt11b^−/−^* embryos at stage 9 (Fig. S5C) and stage 10.5 (Fig. S5C). Expression of a general mid-blastula transition (MBT) marker, *gs17.L*, was unchanged (Fig. S5A,B), consistent with normal progression of early cell cycles. There was variability across replicate samples, consistent with the phenotypic variability (e.g. see M/Z replicate one versus replicate two in Fig. S5B). In addition, analyses of differentially expressed genes showed dysregulation of apparently unrelated genes involved in metabolism, particularly at stage 9, which may be indicative of developmental delay in some metabolic activities (Fig. S5D). Thus, *wnt11b* is strictly, but variably, required maternally for normal dorsoventral patterning and for the normal progression through gastrulation.

To confirm the specificity of these effects, we conducted rescue experiments by injecting oocytes obtained from a *wnt11b^−/−^* F2 female with *wnt11b* mRNA and then fertilising these oocytes using oocyte host-transfer methods (see Materials and Methods). We injected a low dose (20 pg) of transcript to avoid overstimulation of Wnt signalling. Injected and uninjected mutant oocytes were cultured for 24 h, stimulated to mature and then transferred to the body cavity of a wild-type female. After subsequent ovulation, host-transferred eggs were fertilised with sperm from a homozygous mutant male. Embryos derived from mutant oocytes injected with wild-type *wnt11b* exhibited somewhat restored levels of organiser genes and underwent normal gastrulation (Fig. S6; Table 1).

### Phospho-Lrp6 and Dvl regulation are normal in *wnt11b* mutant eggs

Lrp6 activation has been hypothesised to initiate β-catenin stabilisation during axis formation, either in oocytes or as part of signalling endosomes translocated during cortical rotation. We therefore sought to determine the patterns of Lrp6 phosphorylation during early development and to identify the extent to which these patterns were dependent on maternal Wnt11b. We assessed the state of Lrp6 activation in oocytes, eggs and early embryos by immunoblotting against a phospho-epitope of Lrp6, S1490, that is elevated by Wnt ligand binding and essential for the response to Wnt signals (Tamai et al., 2004; Zeng et al., 2005). To generate a more robust and reproducible signal, we injected oocytes with ‘trace’ amounts of exogenous mouse *Lrp6* mRNA (∼20-50 pg).

**Fig. 5.**
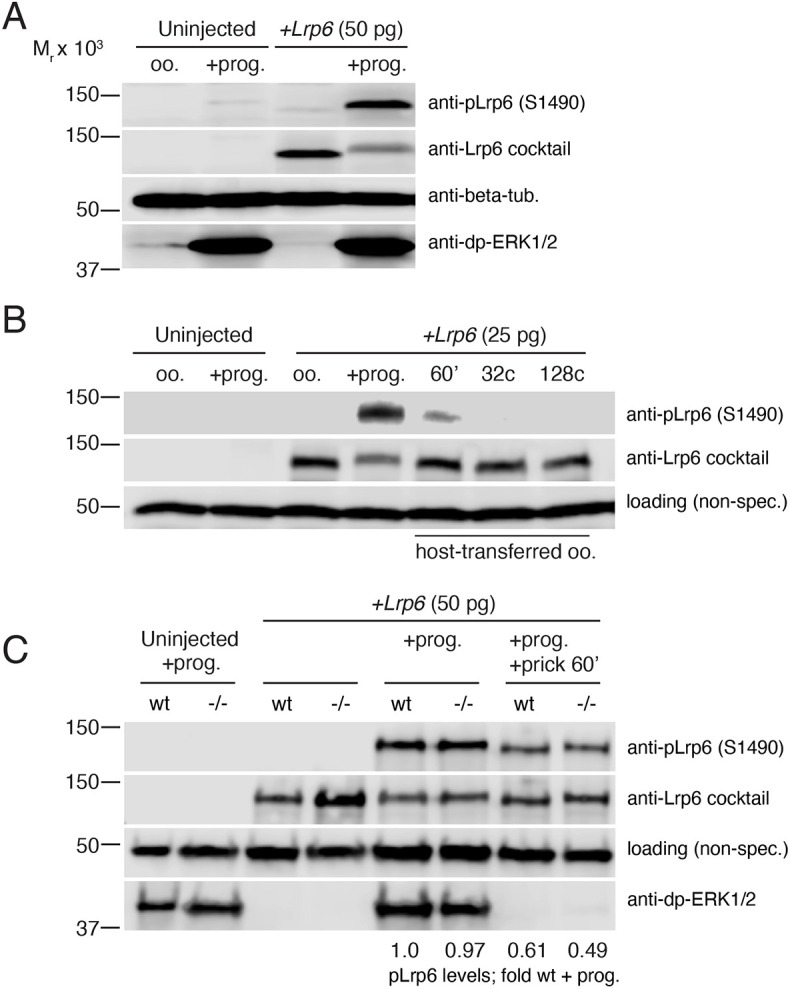
**Wnt11b does not regulate Lrp6 phosphorylation.** (A) Immunoblotting of control oocytes and oocytes injected with mouse *Lrp6* mRNA, untreated (oo.) or treated with progesterone (+prog.). The blot was reprobed using anti-pLrp6 (S1490) and anti-Lrp6 mAbs (anti-Lrp6 cocktail). Tubulin and di-phospho-ERK were used as loading controls. (B) *Lrp6*-injected oocytes were fertilised by host-transfer and blotted against anti-pLrp6 (S1490) and two anti-Lrp6 mAbs (anti-Lrp6 cocktail). A non-specific (non-spec.) band was used to assess equal loading. (C) Immunoblotting of wild-type (wt) and homozygous mutant female (−/−) oocytes, treated with progesterone, without or with prick-activation (+prick 60′). Relative migration (M_r_) of molecular weight standards is on the left. Normalised relative quantification values for pLrp6 in panel C are shown below the blot.

In wild-type samples, S1490 Lrp6 phosphorylation was absent in stage VI oocytes but stimulated in progesterone-treated/mature oocytes (Fig. 5A). Endogenous phospho-Lrp6 was weakly detected in mature oocytes but not in untreated stage VI oocytes. Furthermore, this phosphorylation was transient and was downregulated following either prick-activation or normal fertilisation of host-transferred eggs. Notably, phospho-Lrp6 was substantially reduced by 60 min postfertilisation, when cortical rotation would be occurring, and remained absent in cleavage-stage embryos (Fig. 5B), when β-catenin stabilisation would be occurring. Phenotypic analysis of sibling embryos confirmed that the low doses of *Lrp6* did not induce axial duplication/dorsalisation when expressed in oocytes, whereas higher doses did (Fig. S7), indicating that the trace amounts of mRNA used are below the threshold for strongly activating β-catenin under these conditions.

Analysis of Lrp6 phosphorylation in *wnt11b* mutant oocytes and eggs showed that the patterns of phospho-S1490 stimulation in eggs and subsequent downregulation were identical to wild-type samples (Fig. 5C). These data suggest that Lrp6 is phosphorylated during oocyte maturation in a Wnt11b-independent manner, and that phospho-Lrp6 disappears before cortical rotation is complete and before the crucial period for β-catenin stabilisation (i.e. the 16-to-32-cell stages).

**Fig. 6.**
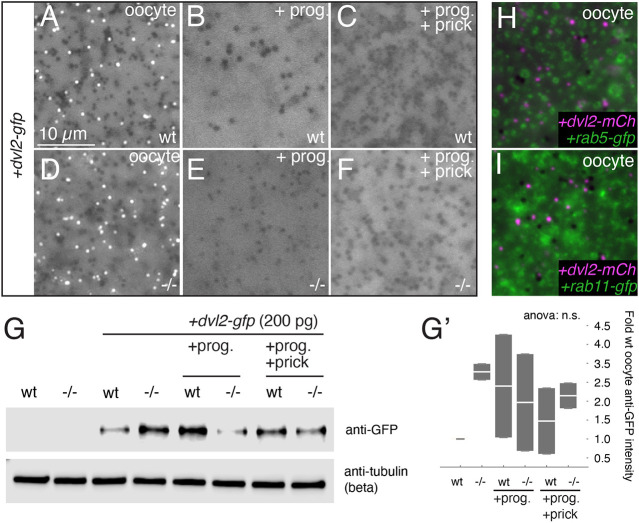
**Wnt11b does not regulate Dvl puncta.** (A-C) Control wild-type (wt) oocytes injected with *dvl2-gfp* and untreated (A), treated with progesterone (2 µM overnight; B) or treated and prick-activated for 60 min (+prick; C). (D-F) Homozygous mutant oocytes treated with or without progesterone and prick-activation. (G) Representative immunoblotting of Dvl2-GFP (anti-GFP) in wild-type (wt) and mutant (−/−) oocytes treated with or without progesterone and prick-activation. The loading control was β-tubulin. (G′) Normalised intensity values for anti-GFP bands (*n*=2 blots), plotted relative to injected wild-type oocytes (wt). The white bar in each box indicates the median value per group; top and bottom of the boxes indicate the 0.25 and 0.75 quantile limits, respectively. (H,I) Control oocytes injected with either *rab5-gfp* (H) or *rab11-gfp* (I) along with *dvl2-mcherry*. Scale bar: 10 µm.

We also tested the working hypothesis that Wnt11b might regulate the formation or activity of Dvl puncta, which are thought to act as dorsal determinants in *Xenopus* (Dobrowolski and De Robertis, 2012; Miller et al., 1999), potentially in association with activated Wnt co-receptor Lrp6 (‘signalosomes’; Dobrowolski and De Robertis, 2012; Miller et al., 1999). We injected transcripts encoding Dvl2-GFP into both wild-type and *wnt11b^−/−^* mutant oocytes. In both cases, we observed numerous Dvl2-GFP puncta using live epifluorescence microscopy (Fig. 6). However, we found that Dvl2-GFP puncta disappeared following oocyte maturation and did not reappear after prick-activation in both wild-type and *wnt11b^−/−^* mutant oocytes (Fig. 6A-F). Immunoblotting against GFP showed comparable levels of fusion protein expression, indicating that the loss of puncta is not the result of Dvl protein degradation (Fig. 6G,G′). In addition, we tested the propensity of Dvl2 puncta to associate with endosomal structures in wild-type *Xenopus* oocytes. However, we failed to observe colocalisation of Dvl2, tagged with mCherry in this case, with exogenous endosome markers Rab5 and Rab11 (tagged with GFP) in coinjected oocytes (Fig. 6H,I).

These data show that Lrp6 is phosphorylated during oocyte maturation independently of maternal Wnt11b function in *Xenopus*. This phosphorylation is downregulated after egg activation/fertilisation and, although reduced and present during cortical rotation, becomes undetectable by the cleavage stages, when β-catenin stabilisation is required to occur. In contrast, β-catenin levels tend to mirror phospho-Lrp6 levels in cultured cell experiments using exogenous Wnt stimulation (Kim et al., 2013; Li et al., 2012). Also, our data show that large aggregates/puncta of Dvl are found in a complementary pattern to Lrp6 phosphorylation and do not localise with endosomal markers in oocytes. Thus, these results generally fail to support models invoking dorsally localised signalosomes in axis specification, although it is possible that a subset of dorsally localised complexes with smaller Dvl oligomers (Ma et al., 2020) are present, but below the limit of detection of methods used here.

### Maternal Wnt11b regulates cortical microtubule assembly and cortical rotation

**Fig. 7.**
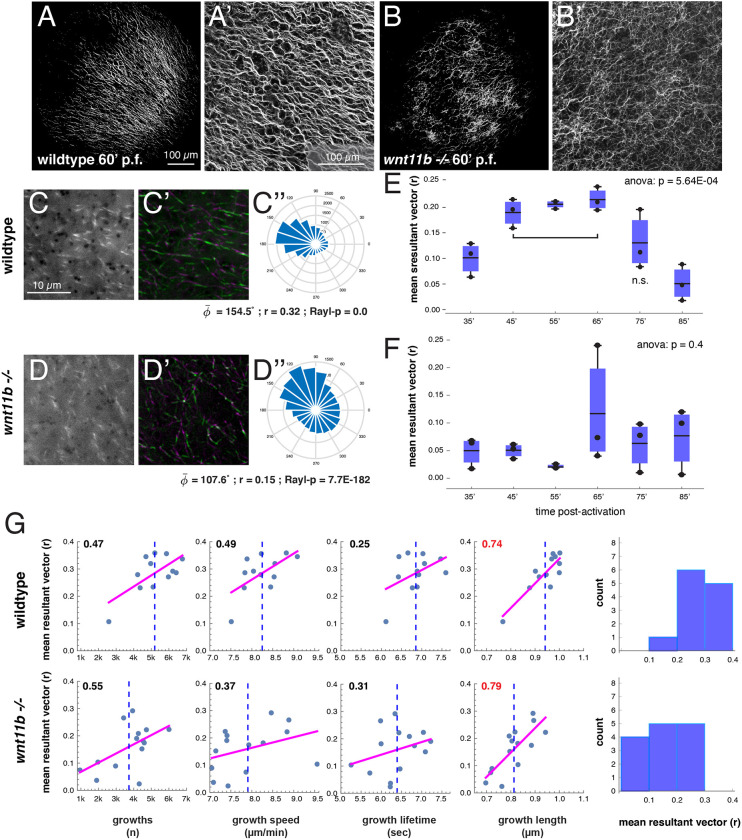
***wnt11b* is required for microtubule alignment during cortical rotation.** (A-B′) Immunostaining of β-tubulin in fertilised wild-type (A,A′) or *wnt11b* mutant (B,B′) zygotes, fixed at 60 min postfertilisation (60′ p.f.). Shown are tiled images of the vegetal surface (A,B) and higher magnification confocal images (A′,B′). (C-D″) Frames from time-lapse movies of prick-activated wild-type (C) and *wnt11b* mutant (D) oocytes injected with *eb3-gfp* mRNA imaged at 60 min post-activation. (C′,D′) Consecutive frame averaging of microtubule motion (green 1-5>magenta 7-11). (C″,D″) Angle histograms of plus end directionality (degree). Circular statistics are listed; φ̅ (phi bar), mean angle, r, mean resultant vector length, Rayl-p, *P*-value of Rayleigh test for circular uniformity. (E,F) Box and whisker plots of mean r-value of prick-activated wild-type (E) and and *wnt11b* mutant (F) oocytes injected with *eb3-gfp* mRNA and imaged at the indicated time points post-activation (35-85 min). The dark bar in each box indicates the mean; the bottom and top edges of the box indicate the 0.25 and 0.75 quantiles, respectively; individual data points are shown. The *P*-values (one-way ANOVA) are at the top; the bracket indicates significance groups (Tukey's HSD criterion); *n*=3. n.s., not significant versus any group. (G) Correlation plots of r versus dynamic parameters and histograms of r. Magenta lines show the least squares fit line, the blue dotted line indicates the sample mean, correlation values are upper left (red colour indicates statistical significance; *P*<0.05). The rightmost panels show histograms of r values obtained for each sample. An r>3.0 is empirically significant in this context. Scale bars: 100 µm (A,A′, also for B,B′); 10 µm (C, also for C′,D,D′).

Because *wnt11b* mutant embryos showed considerable variability with regard to dorsal gene expression and axis formation despite the presence of Lrp6 phosphorylation, we considered whether Wnt11b might be required for the proper distribution of so-called dorsal determinants rather than their generation. To test this idea, we first assessed general microtubule organisation in eggs derived from *wnt11b^−/−^* homozygous mutant females. Eggs from wild-type and mutant females were fertilised (with sperm from either wild-type or mutant males) and fixed during cortical rotation, around 60 and 80 min after fertilisation. Immunostaining against β-tubulin showed that zygotes lacking maternal *wnt11b* (regardless of sperm genotype) exhibited disorganised microtubules (Fig. 7B,B′; Table S2). These remained in a loose/sparse network and did not form full parallel arrays at either time point (0% normal; *n*=48), suggesting that microtubule assembly was not merely delayed in *wnt11b* mutant eggs. Control eggs invariably developed well-formed parallel microtubule arrays at both time points (91% normal; *n*=45) (Fig. 7A,A′; Table S2). Similar results were seen in prick-activated eggs derived from *in vitro*-matured oocytes from wild-type and mutant females analysed at 70 min post-activation (Table S2).

To more accurately quantify the effect of maternal Wnt11b deficiency on microtubule dynamics and on cortical rotation, we performed live imaging of microtubule plus end dynamics. We isolated wild-type and *wnt11b^−/−^* mutant oocytes and injected *eb3* (*mapre3*)*-gfp* mRNA as a microtubule plus end marker. These oocytes were treated with progesterone *in vitro* to induce maturation and then prick activated. Live imaging analyses were performed as previously described (Olson et al., 2015) using oocytes, eggs and prick-activated eggs undergoing cortical rotation (at 60 min postfertilisation).

Plus end dynamics in both oocytes and progesterone-treated oocytes (eggs; *n*=8 each) were comparable with previous reports (Olson et al., 2015) in both wild-type and mutants. Although *wnt11b^−/−^* mutant samples tended to have more plus end growths in oocytes and fewer plus end growths in eggs, as well as more rapid growth speed (Table S3), compared with wild-type cases, none of the differences between groups at the oocyte/unfertilised egg stages were sufficient to reject statistical non-significance. Movies of activated eggs in wild-type control samples during cortical rotation (*n*=12) revealed numerous clearly aligned plus end growths, indicative of robust cortical rotation (Fig. 7C-C″; Fig. S8A-A″; Movie 2). Plus end tracking analyses identified significant concentration around a mean angle (φ̅) in each case, using the Rayleigh test for circular uniformity (Batschelet, 1981; Berens, 2009). In addition, we previously established a mean resultant vector length of r=0.3 as an empirical measure of directionality associated with the establishment of cortical rotation (Olson et al., 2015). This value was chosen because the 95% confidence intervals around the mean angle become asymptotically minimal as r approaches this magnitude. Wild-type activated eggs exhibited an average mean resultant vector length of ∼0.3, with over half the r values above this number (Fig. 7G). Only one sample registered below 0.1, a mean vector length indicative of poor directional organisation (where r=0 would be completely undirected, and r=1 would be uniformly directed on one angle).

Movies of *wnt11b* mutant eggs during cortical rotation (*n*=14) revealed considerable variability in plus end growth (Fig. 7D-D″; Fig. S8B,C; Movies 3 and 4), in line with the variability seen in anti-tubulin immunostaining and in axial development. Some eggs exhibited apparently normal directional growth, whereas others showed randomly directed, disorganised growth. Curiously, plus end tracking analyses showed none of the mutant eggs exhibited a mean resultant vector length>0.3, despite clearly directional growth direction in some cases. Several cases reached a magnitude of r ∼0.25, but many were much lower, resulting in a significantly lower average mean vector length (0.16 compared with 0.29 in controls; Fig. 7G; Table S3).

To determine the extent to which cortical rotation might be precocious or delayed in mutant eggs, we imaged several wild-type and mutant eggs (*n*=3 each) over a time course following prick-activation (∼10 min intervals from 35-85 min; Fig. 7E,F). Whereas wild-type samples exhibit a significant increase in microtubule directionality (i.e. vector length; Fig. 7E), this increase is absent in mutant eggs (Fig. 7F). This result is consistent with the results in the 60 min time point experiments, although we note that the 65 min time point was highly variable in the mutant eggs.

A comparison of microtubule growth parameters during cortical rotation showed that all dynamic parameters were more variable in the mutant eggs, evident in higher coefficients of variation (standard deviation divided by the mean) (Fig. 7G; Table S3). Mean growth speed was similar in both wild-type and mutant eggs at 60 min post-activation, and activated *wnt11b* eggs exhibited fewer plus end growth events in the cortex, as well as shorter growth lifetime and lengths, although only the length difference was sufficient to reject a null statistical hypothesis. Plus end growth speed was also generally correlated with more robust directionality. However, in one mutant sample with higher-than-average growth speed, directionality was compromised (9.5 µm/min, r=0.1), suggesting a possible limit to this relationship (Fig. 7G). Additional analyses of growth parameters showed significant correlation of the mean resultant vector length with plus end growth length in both wild-type and mutant samples (ρ=0.74 and 0.79, respectively; Fig. 7G). Other correlations between mean vector and growth numbers, speed and lifetime were also similar between mutant and controls, but weaker (ρ≤0.6, *P*>0.01), as were correlations of the parameters with each other (Fig. S9A,B). However, other complex or non-linear relationships might exist.

Together, these data show that the earliest detectable consequence of maternal *wnt11b* deficiency is a reduction in the overall organisation and alignment of vegetal cortical microtubules during cortical rotation, leading to less ‘robust’ directional orientation. This disruption is coincident with shorter growth length of microtubule plus ends in the fertilised/activated egg. The data do not address the extent to which this effect is owing to direct or indirect regulation of microtubule dynamics.

## DISCUSSION

We used CRISPR/Cas9 technology to generate a loss-of-function mutation in *X. laevis wnt11b.L*, the only maternally expressed member of the wnt11 family in this species. Our analyses revealed a novel role for maternal Wnt11b as a permissive factor enabling robust microtubule-mediated cortical rotation and axis induction. In addition, we found that maternal Wnt11b is necessary for normal gastrulation morphogenesis, whereas zygotic Wnt11b is necessary for normal left-right asymmetry. Both of these latter findings are in line with the more traditional views of Wnt11 function obtained through antisense-mediated knockdown and dominant-negative experiments. The extent to which Wnt11b might act through a similar molecular activity in all these different contexts is unknown. Wnt11b had been previously suggested to represent the main dorsalising determinant upstream of β-catenin activation in *Xenopus*, whereas our results suggest that its role in this process is likely indirect.

Using targeted CRISPR/Cas9 mutagenesis of *wnt11b.L*, we generated a 13 base pair deletion near the end of exon 1, which resulted in an early in-frame stop codon that was inherited through the germline. This deletion disrupts the normal signal peptide cleavage site and creates a premature stop codon two amino acids after a new (suboptimal) cleavage site. This mutant *wnt11b.L* locus would generate at most a two amino acid peptide (Ser-Pro). Other in-frame stop codons are generated downstream of the initial stop codon, but no potential peptides match known proteins. Based on these sequence predictions, and the lack of mutant transcript activity upon overexpression, we interpreted our results under the premise that this mutation represents a null allele. Further genetic analysis, such as failure to complement deletion alleles, would more conclusively demonstrate nullility.

Premature stop codons can cause transcript degradation through nonsense-mediated mRNA decay. This mechanism is unlikely in the case of the −13 bp deletion, as the premature stop codons are in the transcript 5′ end (Dyle et al., 2020). Also, *wnt11b* RNA persists at normal (or slightly lowered) mRNA levels. A genetic compensation response of related genes can be triggered by nonsense-mediated mRNA decay (El-Brolosy et al., 2019; Ma et al., 2019). Our data do not directly address this phenomenon, but we do note that the related *wnt11.L/S* genes show reduced, rather than elevated, expression, a result inconsistent with compensation.

Nonetheless, also not addressed in this study, a structural or regulatory role may exist for *wnt11b* transcript, based on oligo-mediated transcript degradation experiments in host-transferred embryos (Tao et al., 2005), which exhibit a more severe ventralisation phenotype. Other localised RNAs have non-coding roles in cytoskeletal organisation in *Xenopus* (Kloc, 2009), including *plin2* (*fatvg*), which is required for microtubule organisation and cortical rotation, and therefore axis formation (Chan et al., 2007).

It has long been problematic to reconcile and incorporate various embryological, cell biological and biochemical studies on Wnt signalling and early development into a coherent understanding of the initiating steps in axis specification in *Xenopus* (or indeed in any vertebrate embryo). The data presented here allow several refinements to current models. First, our data suggest that *wnt11b* mRNA is unlikely to be the major axis-inducing agent enriched dorsally by cortical rotation because axis signalling can occur in its absence. High-resolution transcriptomic analyses have also generally failed to identify significant dorsal enrichment or polyadenylation of *wnt11b* (Domenico et al., 2015; Flachsova et al., 2013). Second, we found that Wnt11b signalling is not required for Lrp6 phosphorylation, which is activated during oocyte maturation and then attenuated during egg activation/fertilisation. The presence of phospho-Lrp6 in the egg and its diminution following activation had been noted by Davidson et al. (2009); our observations support this idea, but also show that phosphorylation is stimulated during oocyte maturation. Third, Dvl protein forms visible puncta in oocytes but not in eggs, a pattern complementary to phospho-Lrp6 (and unchanged in *wnt11b* mutants), suggesting that these puncta (as traditionally viewed) are likely unrelated to Lrp6 activation or signalosomes. Furthermore, the disappearance of visible Dvl puncta during oocyte maturation indicates that these (large) aggregates are unlikely to be β-catenin-stabilising agents transported by cortical rotation.

Our data do not rule out a small domain of persistent phospho-Lrp6 and/or small Dvl oligomers at the cell surface (Ma et al., 2020), which might be below the limits of detection in our assays. It is not clear to what extent small Dvl oligomers might behave differently than the larger ‘puncta’ in this context however. Other wnts are not highly expressed maternally, and with our present work, in conjunction with recent evidence for a cytoplasmic role for Huluwa in fish and frogs (Yan et al., 2018), the preponderance of evidence is accumulating that direct β-catenin stabilisation in the early morula is Wnt ligand-independent. Intriguingly, a growing body of evidence shows that regulation of β-catenin in the mammalian AVE occurs independently of secreted Wnt ligand signalling, but does involve Tdgf1, as is the case in frogs as well (reviewed by Houston, 2017).

One implication of these findings is that Wnt11b would be signalling in a para/autocrine fashion, acting on the single-cell egg to modulate microtubule dynamics (directly or indirectly). Recent proteomic data support the idea that Wnt11b protein expression is elevated during oocyte maturation (Van Itallie et al., 2022 preprint; Peshkin et al., 2019 preprint – visualised on Xenbase, Fig. S10) and other studies indicate active translation in the egg (M. D. Sheets, personal communication). This pattern is mirrored by Frizzled (Fzd7), a Wnt11 family receptor, suggesting that this signalling pathway becomes active in the egg. Cortical rotation is a self-organising and variable process, with a wide range of cortical displacements being compatible with normal development (between 13-35° of arc; Vincent and Gerhart, 1987), likely owing to the additional directional transport of so-called dorsal determinants on the parallel microtubule arrays. Because these processes likely generate more dorsal translocation than is strictly needed, cortical rotation is somewhat robust to perturbation. One important function of maternal Wnt11 signalling might therefore be to maintain this robustness by elevating microtubule dynamics above some threshold for efficient self-organisation to occur.

A large body of work has demonstrated the importance of Wnt/PCP signalling mediated by Wnt11 and other ligands in the regulation of gastrulation and axial morphogenesis through the control of convergent extension and other cellular behaviours (Solnica-Krezel, 2005). In this regard, Wnt11-Fzd7 signalling is thought to regulate cortical actin/actomyosin cytoskeletal dynamics (Huebner and Wallingford, 2018), resulting in either increased or decreased cell adhesion/cohesion depending on context (Dzamba et al., 2009; Heisenberg et al., 2000; Kraft et al., 2012; Ulrich et al., 2003, 2005; Winklbauer et al., 2001; Witzel et al., 2006). Our microtubule data show that Wnt11b signalling can have an effect on the cytoskeleton independently of controlling cell motility or cell adhesion (the egg is a single cell). *Wnt11b^−/−^* mutant eggs might thus serve as a useful system in which to study the Wnt/PCP or Wnt/calcium regulation of cytoskeletal dynamics without confounding feedback effects resulting from changes in cell adhesion and/or cell polarity. A preliminary analysis of cytoskeletal organisation failed to identify substantial differences in mutant gastrulae, but there were hints that microtubule and cytokeratin organisation might be more extensive, possibly implicating Wnt11b signalling in reducing or promoting reorganisation of these cytoskeletal systems in different contexts. A more thorough and quantitative analysis of overall cytoskeletal dynamics in these embryos is warranted.

In addition to signalling in the egg, our data also show that maternal Wnt11b is essential for blastopore closure and cell behaviour during gastrulation, in this context potentially through regulation of actomyosin and convergent extension (see above). Maternal *wnt11b* compensates for zygotic loss of *wnt11b* (in F2 homozygous mutants derived from heterozygous crosses), and maternal mRNA injection of *wnt11b* is sufficient to rescue the M/Z phenotype. MZ*wnt11b^−/−^* mutants exhibit a delay in gastrulation, suggesting that other ligands (e.g. *wnt5* homologues) or signals may eventually partially compensate. We note that gastrulation in MZ*wnt11b^−/−^* embryos does appear to progress once zygotic expression of *wnt11* (*wnt11r*) reaches normal levels (∼stage 13) even though *wnt5* homologues are expressed before this time. However, double morpholino-based knockdown of Wnt11b/Wnt11 results in similarly delayed gastrulation, with abnormal extension of the archenteron (Van Itallie, et al., 2022). Thus, low levels of Wnt11 family function (whether through delayed *wnt11* expression in M/Z mutants or through residual protein in knockdowns), with or without Wnt5 function, might be sufficient for ultimate closure of the blastopore. Alternatively, Wnt5 proteins could be sufficient for gastrulation to progress in the absence of Wnt11 family function, but with different (slower) kinetics. Future work, enabled by the *wnt11b^−/−^* mutants described here, will help distinguish these possibilities.

Maternal Wnt/PCP components (*dvl2/3*, *fzd7*, *ptk7*, *gpc4* and *vangl2*) are required for full convergent extension in zebrafish embryos, suggesting a conserved role for Wnt signals near the onset of gastrulation (Jussila and Ciruna, 2017; Xing et al., 2018). Maternal Wnt11b could regulate cell adhesion or cell behaviours before gastrulation, such as those occurring in the vegetal endoderm cells (e.g. vegetal rotation, separation behaviour; Wen and Winklbauer, 2017; Winklbauer and Schürfeld, 1999; Winklbauer et al., 2001), and thus could affect convergent extension or other morphogenetic movements indirectly. Loss of Wnt11b could also alter convergent extension indirectly by elevating BMP signalling and/or expression, which is inversely correlated with convergent extension (Myers et al., 2002) or through regulation of Nodal3.1-FGFR1 signalling (Yokota et al., 2003). A more extensive analysis of signalling interactions and perigastrular cell behaviours in *wnt11b* mutants would help distinguish these possibilities.

Our genetic studies also support the idea of a strictly zygotic role for Wnt11b in the establishment of left-right asymmetry in *Xenopus* (Walentek et al., 2013). F2 homozygous mutants for *wnt11b* derived from heterozygous crosses undergo normal axis formation and gastrulation, and yet a subset develops altered left-right asymmetry (∼25%). A similar proportion is seen in MZ*wnt11b*^−/−^ homozygotes. Taken together with the morpholino-based observations of Walentek et al. (2013), which would affect only zygotic Wnt11b, these data suggest that left-right signalling is a function of zygotic Wnt11b. However, this must be only a biassing signal because roughly half the maternal/zygotic mutants develop bilateral or reduced *pitx2c* expression in the left lateral plate mesoderm – a condition which itself randomises asymmetry, thus potentially accounting for the 25% incidence of abnormal asymmetry.

In the context of left-right asymmetry, previous evidence suggests that Wnt11b controls cilia polarisation and cell shape in the ciliated cells of the posterior notochord/gastrocoel roof plate to establish leftward fluid flow (Schweickert et al., 2007; Walentek et al., 2013). Inhibition of ‘nodal flow’ results in randomised left-right asymmetry in frogs and mice (Nonaka et al., 1998; Schweickert et al., 2010); we would therefore expect abnormal cilia and reduced fluid flow in *wnt11b* mutants. Wnt/calcium signalling was implicated in this aspect of Wnt11b signalling, based on comparative inhibitor studies (Walentek et al., 2013), and this branch of the Wnt network was implicated in tissue separation behaviour as well (Winklbauer et al., 2001). It will thus be interesting to determine the extent to which Wnt11b-mediated Wnt/calcium signalling underlies the multiple roles of Wnt11b during development.

Other proposed functions of Wnt11b during development were not indicated by the genetic data presented here, including roles in pronephros, heart and neural crest development. It is possible that *wnt11* or other functionally related wnts can compensate in these contexts, or that these defects might be secondary to alterations in gastrulation caused by the various loss-of-function reagents.

Overall, it is hoped the development of maternal-effect *wnt11b* mutants (the first such engineered maternal mutation in *Xenopus*) will be valuable for studying Wnt signalling activities within individual cells, as well as in conserved gastrulation movements and cell polarity signalling at the genetic level. Wnt11b mutants could be combined with other tools commonly used in *Xenopus* embryology including morpholino/oligo- or F0 CRISPR-mediated gene knockdowns to further our understanding of diverse processes in early vertebrate development.

## MATERIALS AND METHODS

### *Xenopus* embryos and oocytes

Adult *X. laevis* wild-type J-strain females (RRID:NXR_0024) were induced to ovulate using human chorionic gonadotropin (hCG, MP Biomedicals). Eggs were collected and fertilised in 0.3× Marc's Modified Ringer's (MMR) [10× MMR: 1 M NaCl, 18 mM KCl, 20 mM CaCl_2_,10 mM MgCl_2_ and 150 mM HEPES (pH 7.6)], using a sperm suspension (J-strain wild-type or *wnt11b^−/−^* mutant). Embryos were dejellied at the two- to eight-cell stage in 2% cysteine in 0.1× MMR (pH 7.8) for 4 min before washing the embryos with 0.1× MMR. Embryos were cultured to the desired stage at 18-24°C in 0.1× MMR. For microinjection, fertilised eggs were dejellied 1 h after fertilisation as above and transferred to 2% Ficoll (Pharmacia)/0.3× MMR and injected with 2-10 nl of solution as desired using an air-driven injector (PLI-100; Harvard Apparatus). Injected embryos were washed in 0.1× MMR for 2-4 h/overnight after injection.

Ovary was isolated from anaesthetised wild-type and mutant females and divided into 1 cm segments before storage at 18°C. Oocytes were manually defolliculated in a modified oocyte culture medium [OCM; 67% L-15, 0.05% PVA, 1× Pen-Strep (pH 7.6-7.8); Houston, 2018, 2019] using watchmaker forceps (Dumont #4 or #5) and cultured at 18°C. Alternatively, for *in situ* hybridisation, oocytes were enzymatically defolliculated by treatment with 0.02% Liberase™ (Roche Applied Science) for 1-1.5 h in 0.5× Dulbecco's PBS with rocking, followed by extensive washing in OCM.

Animal work was conducted in compliance with protocols established by the Institutional Animal Care and Use Committees (IACUCs) of the University of Iowa and the Marine Biological Laboratory.

### Oocyte host transfer

Oocytes (*wnt11b*^−/−^ or wild type) were injected in the vegetal pole with doses of RNAs indicated in the text and cultured for 24 h at 18°C before being matured by treatment with 2 μM progesterone. Matured oocytes were colored with vital dyes, transferred to egg-laying host females, recovered and fertilised as previously described (Heasman et al., 1991; Houston, 2018, 2019).

### CRISPR/Cas9 mutagenesis

Mutations in *wnt11b.L* were generated in F0 embryos using CRISPR/Cas9 RNP injection. Guide RNAs were designed against exons 1-2 of *X. laevis* (J-strain) *wnt11b.L* (Table S1). *Wnt11b.L* is the only homologue of Wnt11b, and is the only member of the family to be expressed maternally (Session et al., 2016). *In vitro*-transcribed guide RNAs were made from PCR-generated templates (Bhattacharya et al., 2015) and were complexed with Cas9 protein (CP01; PNA Bio) at 37°C before injection into the animal pole of fertilised eggs at the one- to two-cell stage. Mutagenesis was verified by sequencing of PCR products using DNA obtained through genotyping of whole embryo or tail samples, or through biopsy of the foot webbing of post-metamorphic animals. DNA isolation was performed as in Bhattacharya et al. (2015) and sequencing was performed at the Marine Biological Laboratory (MBL; MA, USA) or at the Carver Center for Genomics (University of Iowa, IA, USA). A founder female was identified that transmitted a 13 base pair deletion in exon 1 at the sgT1 site (chr8L:21220561..21220583) and was used to generate heterozygous male and female F1 offspring. These were interbred by *in vitro* fertilisations to generate homozygous F2 *wnt11b.L* mutant animals (RRID:NXR_2112). Breeding and colony maintenance were carried out by the NXR at MBL.

### Plasmids

Full-length cDNAs for *wnt11b.L* were amplified from wild-type or *wnt11b^−/−^* mutant oocyte total RNA using a high-fidelity polymerase (Q5, New England Biolabs). PCR products were cloned into pCR8/GW/TOPO (Invitrogen) and individual clones were verified by sequencing. Primer sequences are presented in Table S1. Desired clones in the (correct) 5′L1-3′L2 orientation were inserted via recombination into a pCS2+ Gateway-converted vector (Custom Vector Conversion Kit; Invitrogen). Template DNAs for sense transcripts were prepared from *wnt11b/pcs2+* plasmids by *Not*I digestion. Capped messenger RNA was synthesised using SP6 mMessage mMachine kits (Ambion). *Eb3-gfp/pcs2+* RNA was similarly prepared as previously described (Olson et al., 2015). Mouse *Lrp6* in pβ/RN3P was used as previously described (Kofron et al., 2007), prepared by *Sfi*I/T3 digestion and transcription. RNAs for zebrafish *dvl2-mcherry*, *rab5-gfp* and *rab11-gfp* were gifts from D. Slusarski (The University of Iowa, IA, USA).

### Analysis of gene expression using real-time RT-PCR

Total RNA was prepared from oocytes and embryos (pools of three per sample) using proteinase K digestion followed by treatment with RNase-free DNase as described (Oh and Houston, 2017). Real-time RT-PCR was carried out using the LightCycler™ 480 system (Roche Applied Science). Samples were normalised to *ornithine decarboxylase* (*odc1*) or to the geometric mean of *odc1* and *fgfr1*, and relative expression values were calculated against a standard curve of control cDNA dilutions. Samples lacking reverse transcriptase in the cDNA synthesis reaction failed to give specific products. Primer sequences are listed in Table S1. Charts were generated directly from the text file output of the Roche LightCycler 480 software using a custom Python script.

### Whole-mount *in situ* hybridisation

Whole-mount *in situ* hybridisation was performed essentially as previously described (Kerr et al., 2008; Sive et al., 2000). Template DNAs for *in vitro* transcription were prepared by digestion, followed by transcription, with appropriate restriction enzymes and polymerases: *wnt11b/pcs2+* (*Sal*I/T7), *nodal3.1* (from R. Harland, University of California, Berkeley, CA, USA; *Eco*RI/T7), *eomes* and *myod1* (from J. Gurdon, Gurdon Institute, Cambridge, UK; *Eco*RI/T3 and *Bam*HI/SP6, respectively), *sox17a* (from A. Zorn, Cincinnati Children's Hospital, OH, USA; *Asp*718/T3), *pitx2c/pbluescript* (a gift from M. Blum, University of Hohenheim, Stuttgart, Germany; *Not*I/T7) and *sizzled/pcs2+* (from M. Kirschner, Harvard Medical School, MA, USA; Addgene plasmid 16688, *Bam*HI/T3), *wnt8a/pcs2+* (XE10, from R. Moon, University of Washington School of Medicine, WA, USA; Addgene plasmid 16865; *Bam*HI/T3). Antisense RNA probes labelled with digoxygenin-11-UTP (Roche) were synthesised using polymerases and reaction buffers from Promega. Processing of *in situ* hybridisation was performed manually or using a robotic system (Biolane, Intavis).

### RNA-sequencing

Total RNA from two biological replicates of stage 9 and stage 10.5 embryos (three embryos per sample) was quality-tested using an Agilent Bioanalyzer and quantified by fluorimetry. Library preparation for Illumina^®^ NovaSeq 6000 sequencing was performed by the University of Iowa, Department of Biology, Carver Center for Genomics and sequenced by the University of Iowa, Carver College of Medicine, Iowa Institute for Human Genetics, Genomics Division. Raw paired-end reads were processed using the CSBB-v3.0 pipeline (Fortriede et al., 2020; https://github.com/praneet1988/Computational-Suite-For-Bioinformaticians-and-Biologists). Briefly, reads were mapped to the *Xenopus laevis* genome version 9.2 (Session et al., 2016) using *bowtie2* and quantified using *RSEM* (Li and Dewey, 2011). Top differentially expressed genes across both stages 9 and 10.5 were identified using *edgeR* and *DeSeq2* (Love et al., 2014; Robinson et al., 2010) (Fig. S5). The GEO accession number for this study is GSE195806.

### Immunoblotting

Samples were lysed in cell lysis buffer [150 mM NaCl, 20 mM Tris-HCl (pH 7.4), 1 mM EDTA, 1 mM EGTA (pH 8.0), 1% Triton X-100, with protease and/or phosphatase inhibitors] and clarified by centrifugation (10 min at 10,000 ***g***). Lysates were heated in sample buffer (Laemmli, 1970) and the equivalent of 0.5-3 oocytes/embryos were electrophoresed on SDS-PAGE TGX AnyKD Ready Gels (Bio-Rad) or large homemade gels and transferred to nitrocellulose (Power Blotter, Thermo-Pierce). For anti-phospho-LRP6 analysis, samples were lysed fresh (without freezing), and anti-phospho-LRP6 was used first, before stripping and reblotting.

Membranes were blocked in 5% bovine serum albumin (BSA) in TBS, 0.1% Tween 20 (for anti-phospho-LRP6) or 5% nonfat dry milk (HyVee) in PBS, 0.1% Tween 20, and incubated in primary antibody overnight at 4°C. Detection was performed using anti-mouse or -rabbit secondary antibodies conjugated to peroxidase (1:10,000, Jackson ImmunoResearch, 115-035-003 and 111-035-003, respectively) and Licor reagents and equipment (C-Digit Scanner). Quantification of bands was carried out using Image Studio Digits v.5.0 (Licor).

Antibodies and dilutions used were: rabbit anti-phospho-LRP6 polyclonal antibodies [S1490, 1:500, Cell Signaling Technology (CST); RRID:AB_2139327], anti-LRP6 rabbit mAbs (C5C7 and C47E12, 1:500 each, mixed together, CST; RRID:AB_2139329 and RRID:AB_1950408), anti-β-tubulin [mAb E7, 1:1000 concentrate, Developmental Studies Hybridoma Bank (DSHB); RRID:AB_528499], anti-GFP (1:1000, clones 7.1 and 13.1, Roche; RRID:AB_390913) and diphospho-ERK-1 and ERK-2 (1:4000, clone MAPK-YT, Sigma-Aldrich; RRID:AB_477245). In this study, anti-phospho-LRP6 recognised endogenous *Xenopus* and injected mouse phospho-S1490 Lrp6, whereas the non-phospho mAbs recognise mouse but not frog Lrp6. Weaker reactivity of the anti-LRP6 rabbit mAbs against phospho-Lrp6 suggests that phosphorylation may block the relevant epitopes.

### Immunostaining

Whole-mount immunostaining was performed on embryos fixed in MEMFA and stored in 100% methanol (embryo stages), or fixed directly in methanol (eggs), using modifications of previously described methods (Cuykendall and Houston, 2009; Elinson and Rowning, 1988). Fixed samples were rehydrated gradually to 1× PBS, then to PBT [PBS/0.5% Triton X-100/0.2% BSA (fraction V)] and then blocked for 2 h at room temperature in PBT/2.5% BSA. Samples were washed for 1 h the next day in PBT and incubated with primary antibodies diluted in PBT overnight at 4°C with rocking, followed by five 1 h washes in PBT. Incubation with secondary antibodies diluted in PBT was carried out for 2 h followed by washing as above.

The primary antibody used was: anti-β-tubulin mAb E7 (1:200 dilution of monoclonal antibody concentrate; DSHB Hybridoma Product E7, deposited with the DSHB by M. Klymkowsky; Chu and Klymkowsky, 1989; RRID:AB_528499). The secondary antibody used was: goat anti-mouse Alexa-488, diluted in PBT (1:500, Invitrogen/Molecular Probes, A28175). Fluorescence was visualised on a Leica DMI4000B inverted microscope using 20×-63× dry objectives (Leica Microsystems). For confocal analysis, samples were imaged on an SP8 confocal imaging system (Leica Microsystems) using a 20× objective with or without tile-scanning to visualise the entire vegetal surface. Additional immunostaining methods for experiments in Fig. S3 are presented in the supplementary Materials and Methods.

### Time-lapse image analysis of microtubule plus end dynamics

Samples were imaged at room temperature on an inverted, wide-field epi fluorescence microscope (DMI4000B, Leica Microsystems) using an oil-immersion Leica 100×/1.30 N.A. PLANAPO objective. Image acquisition was carried out using a Leica DFC3000G monochrome camera at a frame rate of 2 s per frame and using Leica Application Suite (LAS) X software (v3.7.3). The pixel size was 0.0536×0.0536×0.200 mm and the image size was 1296×966 pixels. Leica files (.lif) were imported directly into MATLAB_2021a using u-track (v2.3; https://github.com/DanuserLab/u-track; Applegate et al., 2011; Jaqaman et al., 2008; Matov et al., 2010) and processed using similar parameters as previously described (Olson et al., 2015). Custom code was inserted to incorporate code from the CircStat circular statistics toolbox (Berens, 2009) into the u-track analysis (https://github.com/houston-lab/paper-code-files). Statistical analyses were performed using MATLAB; the *fdr_bh* package was used for adjusted *P*-values (https://www.mathworks.com/matlabcentral/fileexchange/27418-fdr_bh; Benjamini, 2010; Benjamini and Hochberg, 1995). Consecutive frame averaging was performed using the Time-lapse Series Painter for FIJI (van der Vaart et al., 2011). Images for publication were prepared in Adobe Illustrator and Photoshop using only level and contrast adjustments applied over the entire image. Other modifications included resizing, changing stroke/fill weights and colours and annotated overlays.

### Methodology and statistics

Experiments were repeated at least twice with different mutant frogs or wild-type siblings, typically relying on 20 samples per experiment (which carries more than enough statistical power to identify a perturbation in ∼60% of the samples).

## Supplementary Material

Supplementary information

Reviewer comments
