# Supplementary material for: Maternal Wnt11b regulates cortical rotation during Xenopus axis formation: analysis of maternal-effect wnt11b mutants
Source: Development. 2022 Sep 9;149(17):dev200552. doi: 10.1242/dev.200552 (PMC9515810; doi:10.1242/dev.200552)
Supplement: Supplementary information [file develop-149-200552-s1.pdf]

Supplementary Materials and Methods

Immunostaining

Whole-mount immunostaining on embryos was performed by fixing in MEMFA or 100% methanol (for anti-tubulin and anti-cytokeratin mAbs), followed by storage in methanol. Samples were rehydrated gradually to 1x PBS, then to PBT(PBS/0.5% Triton X-100/0.2% BSA (fraction V)) and then blocked for two hours at room temperature in PBT/2.5% BSA. Primary antibodies (1:5) or Alexa-488 phalloidin (1:500) were diluted in the blocking solution and incubation was carried out overnight at 4˚C. After extensive washing (5 x one hour in PBT), peroxidase- or Alexa-488 conjugated secondary antibodies (1:500; peroxidase goat anti-mouse IgG; Jackson Immunoresearch; or Alexa-488 goat anti-mouse IgG, ThermoFisher) were added where appropriate and incubated overnight at 4˚C. Following washes as above, samples with peroxide secondaries were first washed in PBS lacking detergent, and then incubated in either FITC- or Rhodamine-tyramide diluted 1:100 in a stable peroxide buffer (ThermoFisherPierce). Tyramides were synthesised according to a Xenbase.org wiki protocol and (Hopman et al., 1998). The tyramide detection reaction was carried out for 30 minutes and samples were extensively washed in PBT, including an overnight wash at 4˚C.

For two-colour detection, the initial peroxidase was inactivated by incubation in 2% H_2O_2 in PBS (30 minutes), followed by washing, blocking and antibody incubation as above (peroxidase goat anti-mouse IgM was used for Tor70 detection). Tyramide detection was again performed using the second colour. For final imaging, samples were gradually transitioned to ~50% TDE (2,2′-thiodiethanol)/PBS and visualised using epifluorescence microscopy.

Primary antibodies were 1:5 dilutions of monoclonal antibody supernatants (DSHB Hybridoma Product 1h5, deposited with the DSHB by M. Klymkowsky; (Klymkowsky et al., 1987), RRID:AB_528323; DSHB Hybridoma Product 12/101, deposited with the DSHB by J.P. Brockes; (Kintner and Brockes, 1984), RRID:AB_531892; DSHB Hybridoma Product tor70/Notochord Ab1, deposited with the DSHB by R.M. Harland; (Bolce et al., 1992), RRID:AB_2722485; and DSHB Hybridoma Product XAN-3 (Clone 6F11), deposited with the DSHB by D.S. Sakaguchi; (Lamb et al., 1993)) and 1:200 dilution of concentrated anti-Beta-tubulin mAb E7 (1:200 dilution of monoclonal antibody concentrate; DSHB Hybridoma Product E7, deposited with the DSHB by M. Klymkowsky; (Chu and Klymkowsky, 1989); RRID:AB_528499).

Development • Supplementary information

Supplemental References

Bolce, M. E., Hemmati-Brivanlou, A., Kushner, P. D. and Harland, R. M. (1992). Ventral ectoderm of Xenopus forms neural tissue, including hindbrain, in response to activin. *Development* 115, 681–8.

Chu, D. T. and Klymkowsky, M. W. (1989). The appearance of acetylated alpha-tubulin during early development and cellular differentiation in Xenopus. *Developmental Biology* 136, 104–117.

Hopman, A. H. N., Ramaekers, F. C. S. and Speel, E. J. M. (1998). Rapid Synthesis of Biotin-, Digoxigenin-, Trinitrophenyl-, and Fluorochrome-labeled Tyramides and Their Application for In Situ Hybridization Using CARD Amplification. *J Histochem Cytochem* 46, 771–777.

Kintner, C. R. and Brockes, J. P. (1984). Monoclonal antibodies identify blastemal cells derived from dedifferentiating limb regeneration. *Nature* 308, 67–9.

Klymkowsky, M. W., Maynell, L. A. and Polson, A. G. (1987). Polar asymmetry in the organization of the cortical cytokeratin system of Xenopus laevis oocytes and embryos. *Development* 100, 543–557.

Lamb, T. M., Knecht, A. K., Smith, W. C., Stachel, S. E., Economides, A. N., Stahl, N., Yancopolous, G. D. and Harland, R. M. (1993). Neural Induction by the Secreted Polypeptide Noggin. *Science* 262, 713–718.

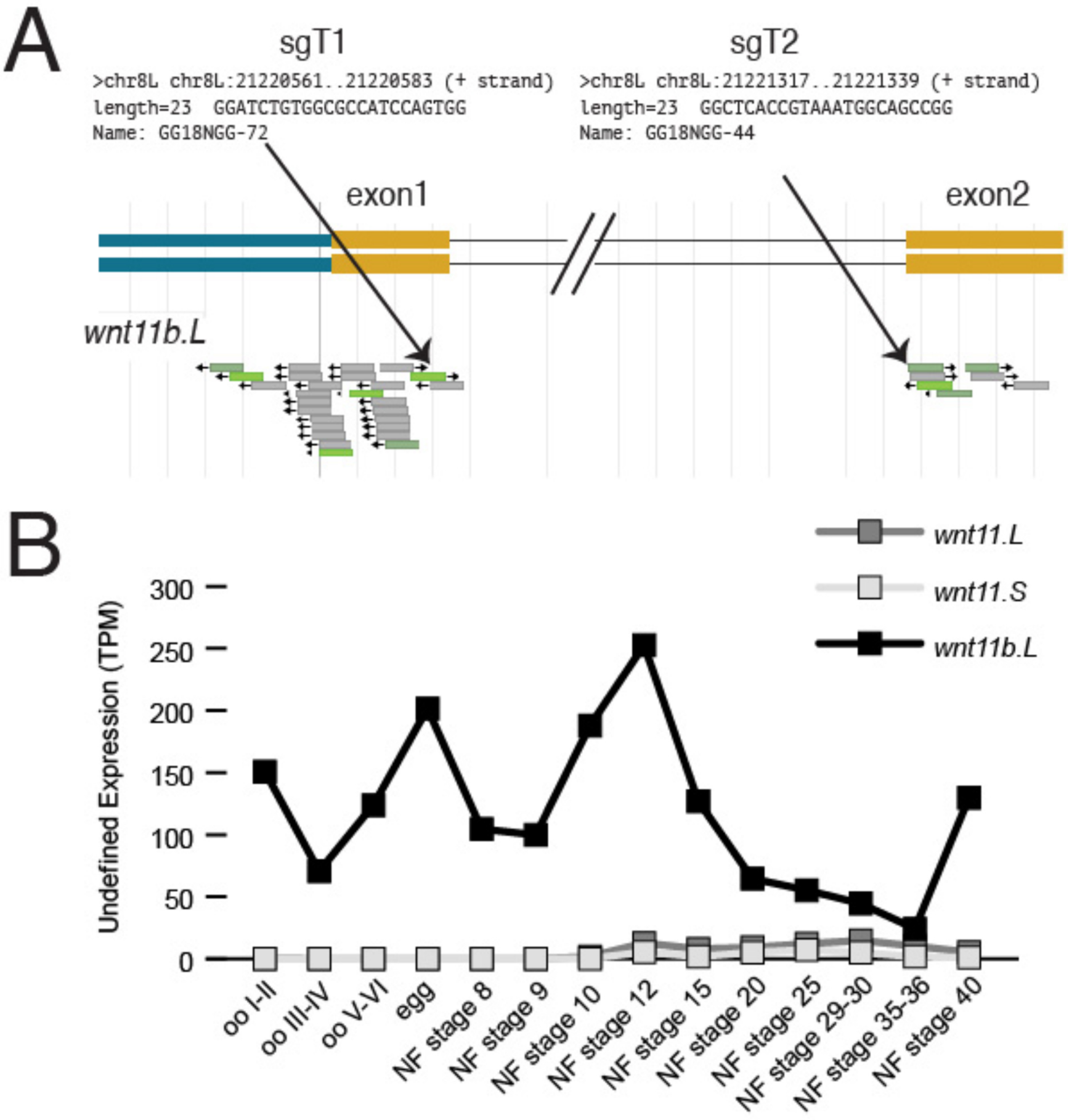

Fig. S1. (A) Location and sequence information for sgRNAs against *wnt11b.L.* Genome browser view was obtained from Xenbase. (B) Expression of *wnt11b.L, wnt11.L* and *wnt11.S* during oogenesis and development. RNAseq expression data from Session et al. (2016) retrieved from Xenbase TPM=transcripts per million; oo=oocyte stage; NF= Nieuwkoop and Faber (1956) stage.

Development • Supplementary information

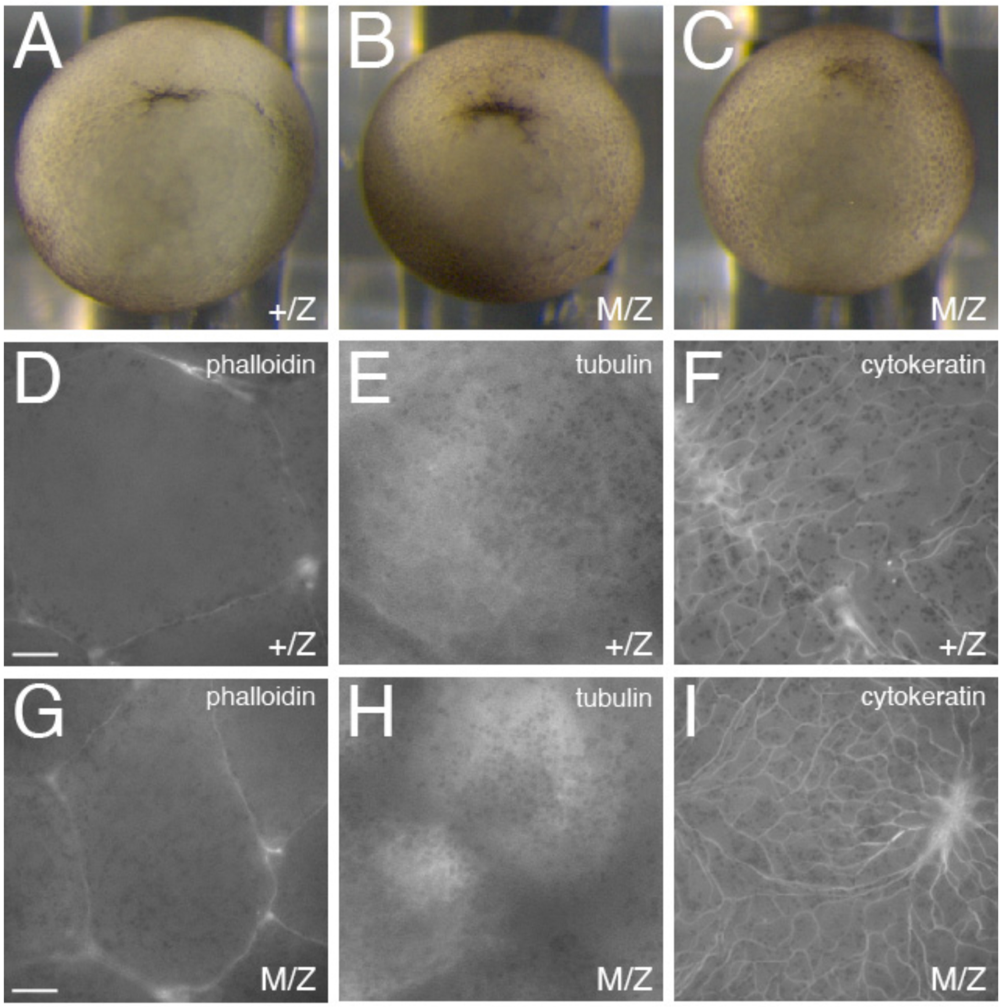

Fig. S2. (A-C) Examples of an *+Zwnt11b+/-* embryo (A) and *MZwnt11b-/-* embryos (B-C) at the onset of gastrulation (NF stage 10-10 1/4), showing variation in timing of dorsal lip formation. (D-1) Immunostaining of against F-actin, beta- tubulin and cytokeratin in heterozygous +/Z (D-F) or *wnt11b* mutant (G-1) gastrulae (stage 10). Equatorial images are shown (63x mag.), scale bars are 10 μm.

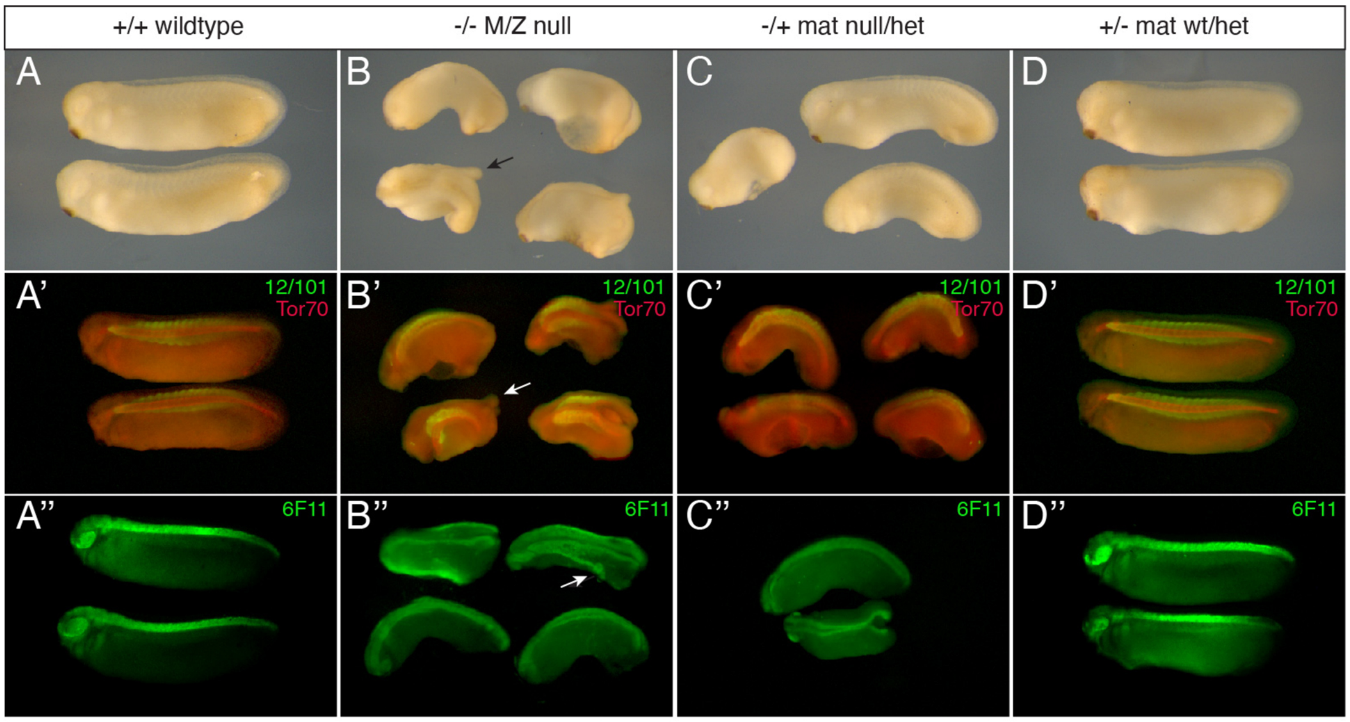

Fig. S3. (A-D) Phenotypes of control (A. D) and maternal-effect *wnt11b* mutant embryos (B, C). (A'-D') Double immunostaining against somite (12/101; green) and notochord (Tor70; red) antigens. (A"-D") Immunostaining against a neural-specific antigen (6F11 (NCAM); green). Arrows indicate ectopic tailbud-like structures.

Development • Supplementary information

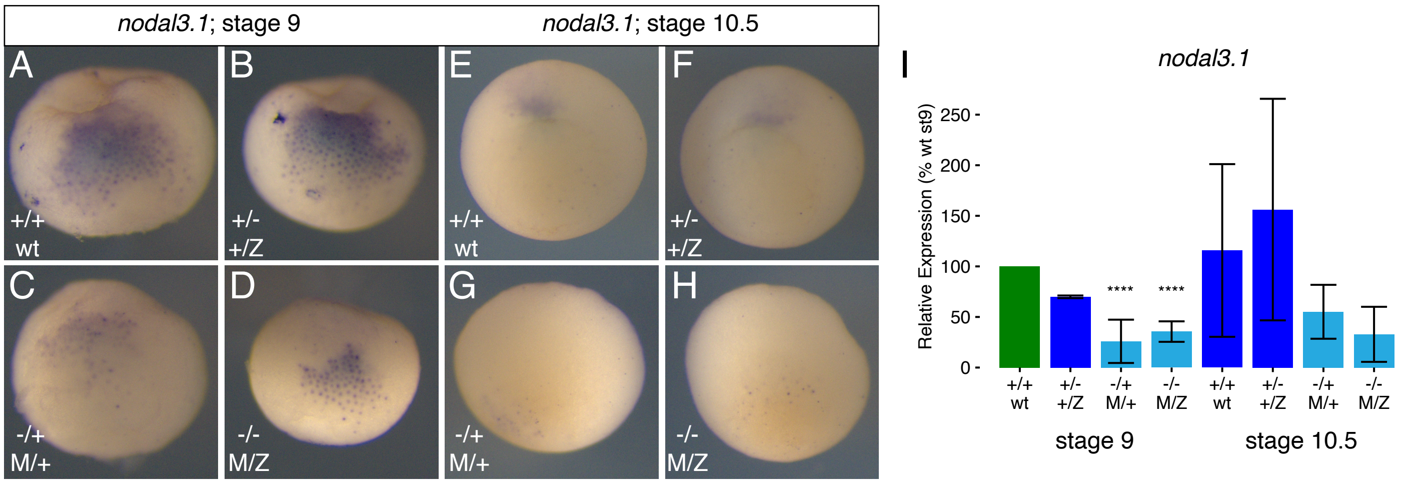

Fig. S4. (A-H) In situ hybridization against *nodal3.1* in maternally wildtype (wt) (A-B, E-F) and mutant *wnt11b* embryos (C-D, G-H) at stage 9 (A-D; dorsal views) and stage 10.5 (E-H; vegetal views, dorsal to top). (I) Real-time RT-PCR analysis of *nodal3.1* at stage 9 and 10.5. The bar in green indicates the sample used for normalization of relative expression. Error bars represent standard deviation of two biological replicates. **** $p < 0.0001$ versus wildtype within stage group.

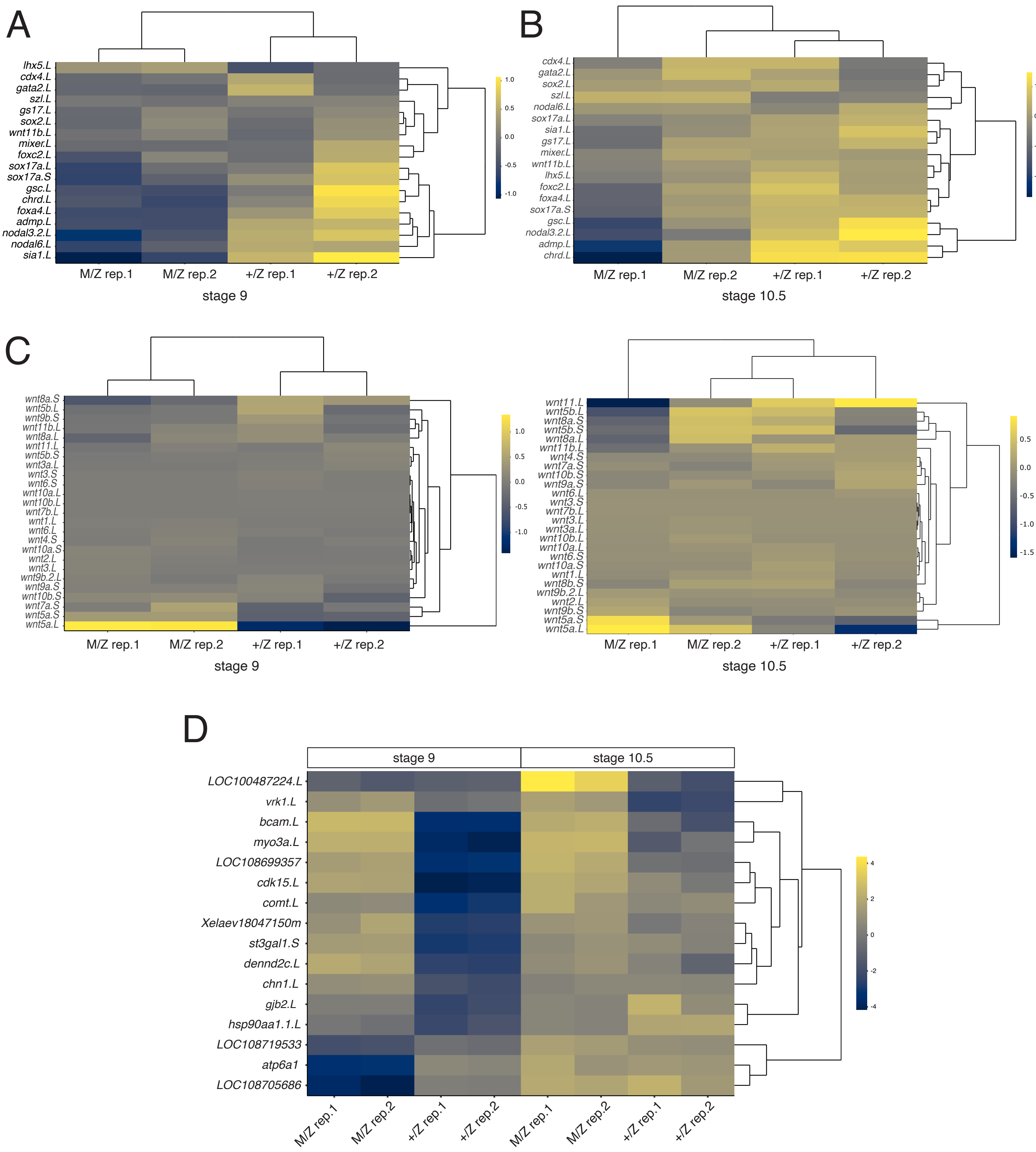

Fig. S5. Heatmaps showing relative gene expression changes for selected germ layer and patterning markers at stage 9 (A) and stage 10.5 (B), stage 9 and 10.5 wnt genes (C) and top 16 differentially expressed genes across both stages (D). Values for each gene represent the differences from the mean value all samples, following regularized log2 transformation of counts. Replicates are biological replicates of three pooled embryos per replicate sample. Darker colors indicate relative downregulation; lighter colors indicate relative upregulation.

Development • Supplementary information

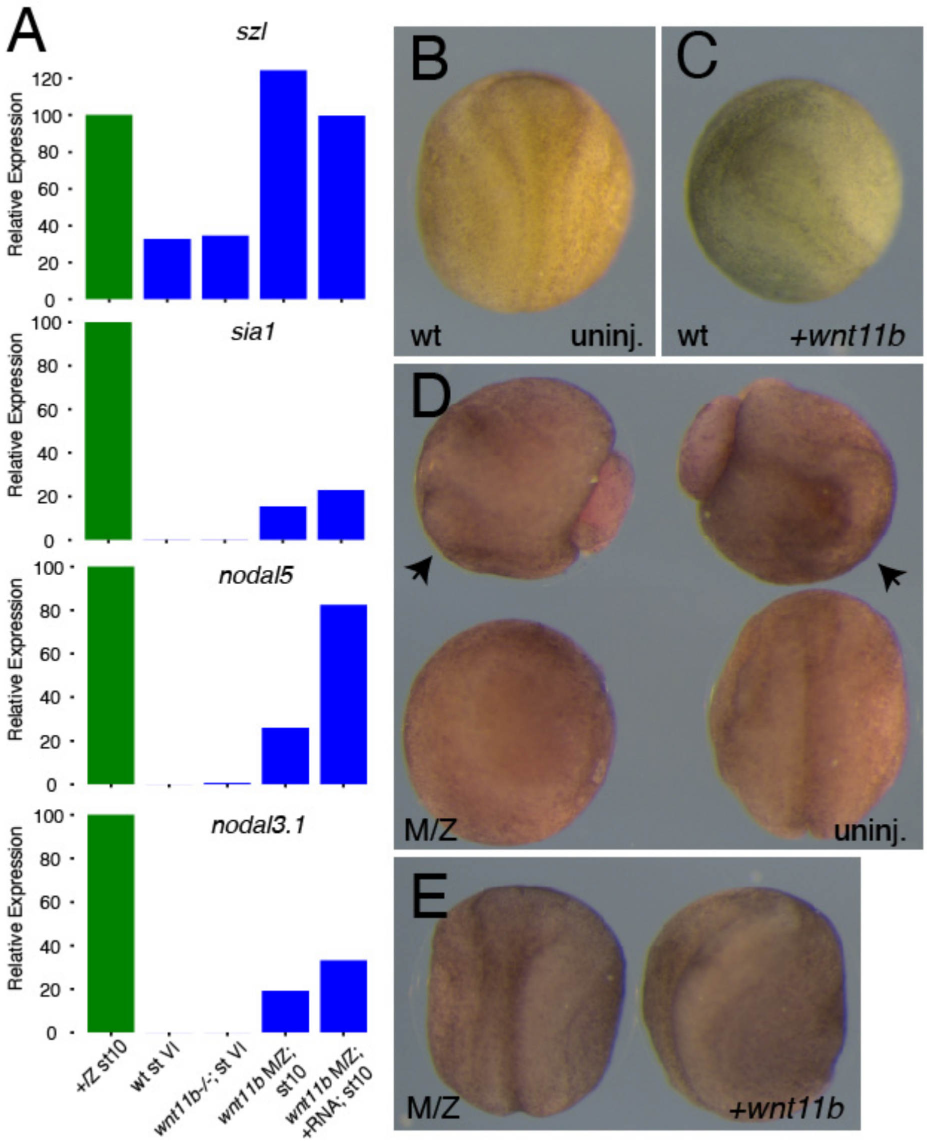

Fig. S6. (A) Real-time RT-PCR analysis of ventral (sz and dorsal (*sia1, noda/5, noda/3.1)* in stage 1O embryos derived from wild type (wt) or *wnt11b* mutant (M/Z) oocytes fertilized by wild type sperm following host-transfer (three pooled oocytes/embryos per sample). Control heterozygous (+/Z) embryos were used for normalization of relative expression (green bar). (B-E) Representative phenotypes of control (+/Z; B, C) and *wnt11b* mutant (D, E) embryos obtained by host-transfer. Samples in B and D were left uninjected (uninj.); samples in (C) and (E) were injected as oocytes with 20 pg *wnt11b* mRNA. Dorsal views, anterior is up except top row in (D), where arrowheads indicate anterior.

Development • Supplementary information

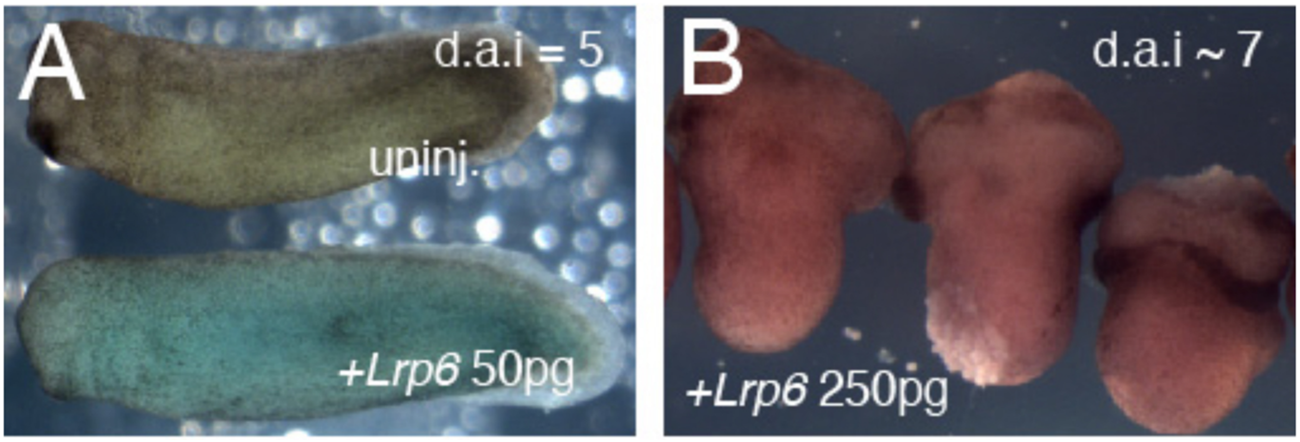

Fig. S7. (A) Representative phenoptyes of embryos derived from host-transferred oocytes left uninjected (uninj.) or injcted with low (A, 50 pg) or high doses (B, 250 pg) of mouse *Lrp6* mRNA. d.a.i, dorsoanterior index (Kao and Elin- son, 1988) of the examples shown.

Development • Supplementary information

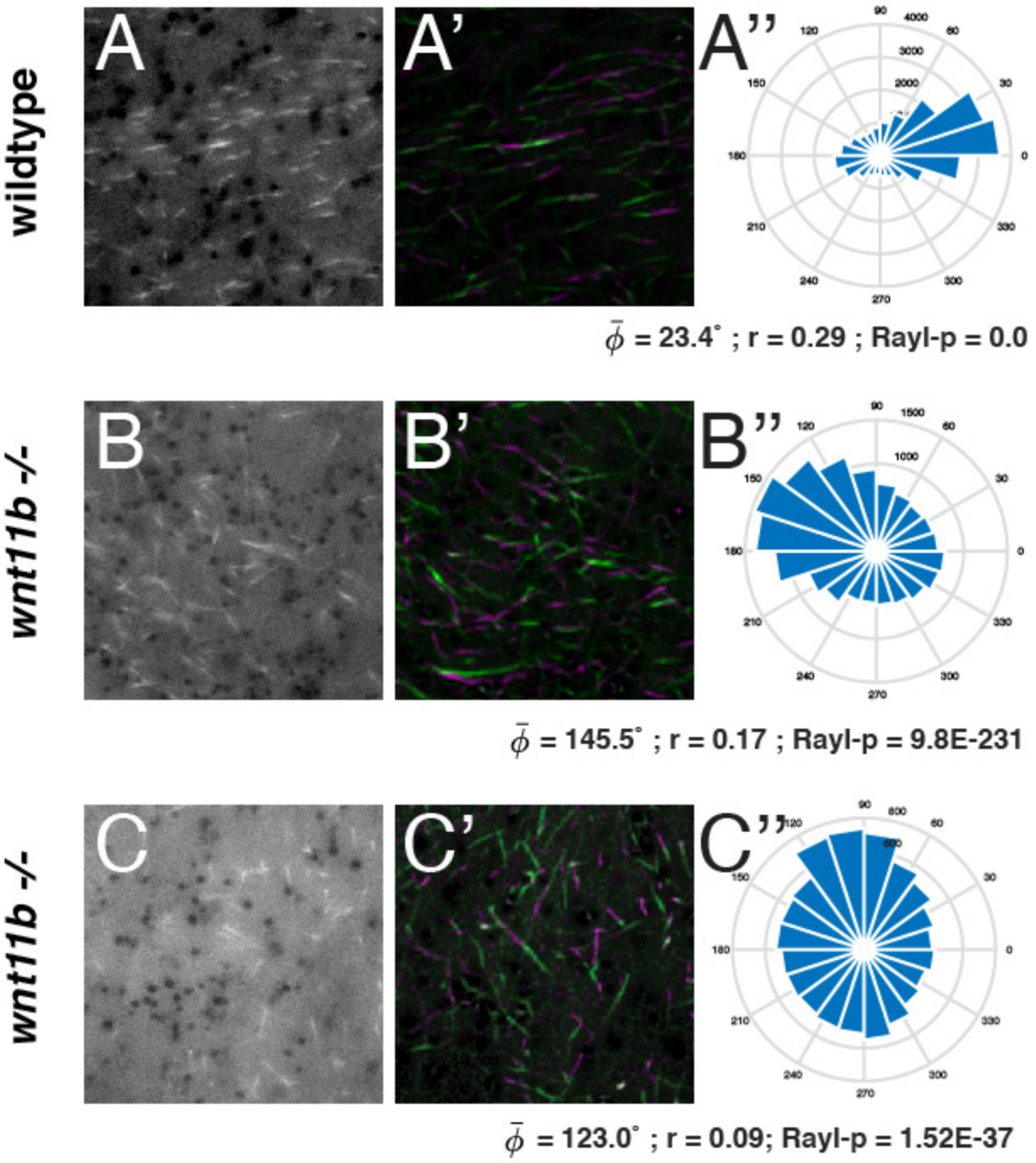

Fig. S8. Additional microtubule tracking examples. (A-C) Representative single frames from time-lapse movies of prick-activated control (A) or *wnt11b-l-* mutant oocytes (B,C) injected with *eb3-gfp* mRNA. (A'-C') Microtubule motion is depicted by averaging frames 1-5 and frames 7-11 and merging pseudo-colored images green and magenta, respectively. (C-C") Angle histogram plots showing individual plus end track directionality (degree) per bin per two minute movie. An example of a *wnt11b-l-* mutant sample with lower directionality (r <0.1) is shown in C-C". Circular statistics are listed; cp (phi bar) = mean angle, r = mean resultant vector length, Rayl-p = p-value of Rayleigh test for circular uniformity.

Development • Supplementary information

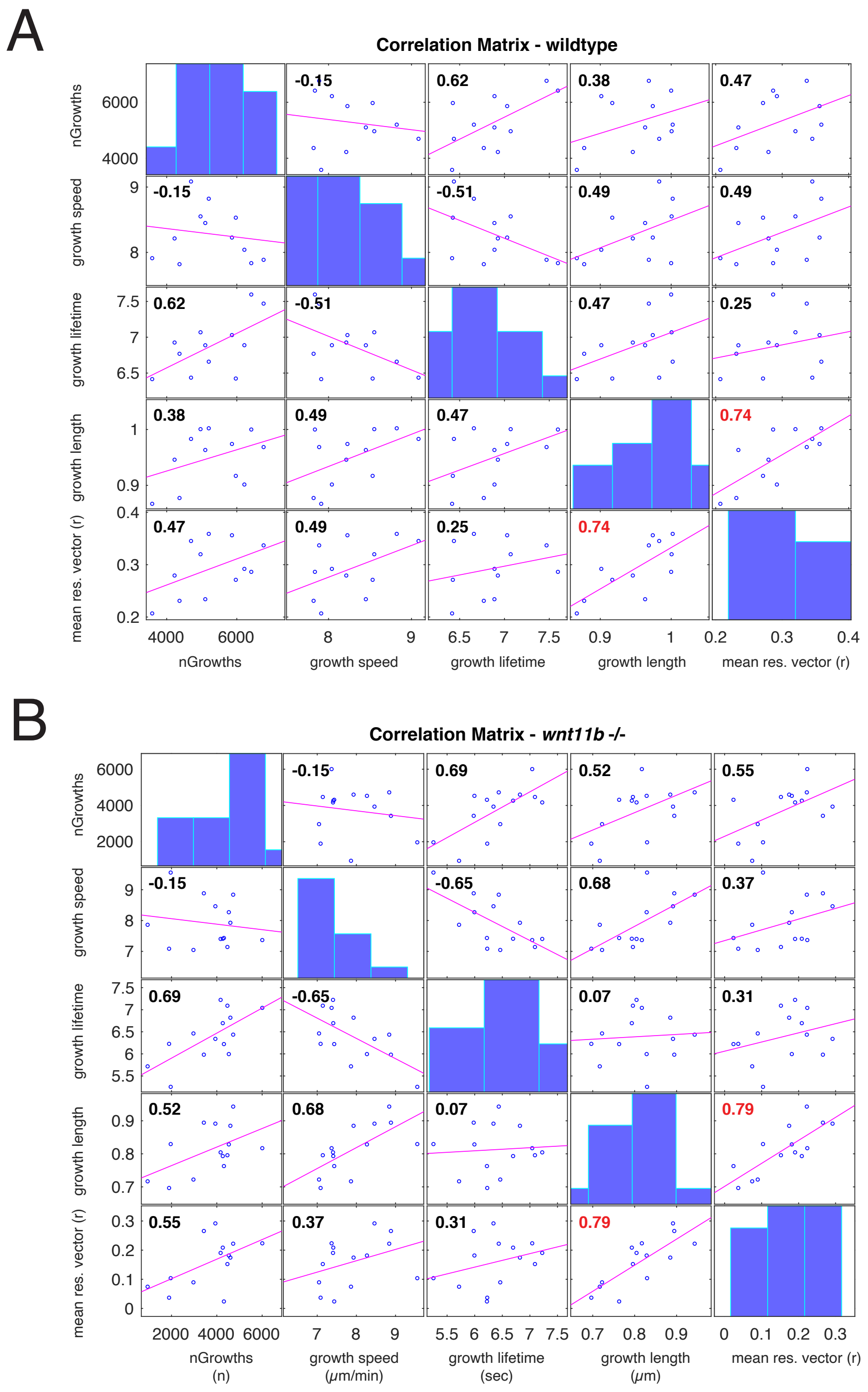

Fig. S9. Correlation matrices and histograms (diagonals) of microtubule dynamic parameters in time-lapse movies from wildtype (A) and mutant (B), prick-activated eggs imaged at 60 minutes post-fertilization. Magenta lines show the least squares fit line, correlation values are upper left (red colour indicates statistical significance; P<0.05). Please note the differing scales between the two matrix plots and the scales in Fig. 7G; the bottom row of each panel is identical to the corresponding panels in Fig. 7G (except for scale and aesthetic adjustments).

Development • Supplementary information

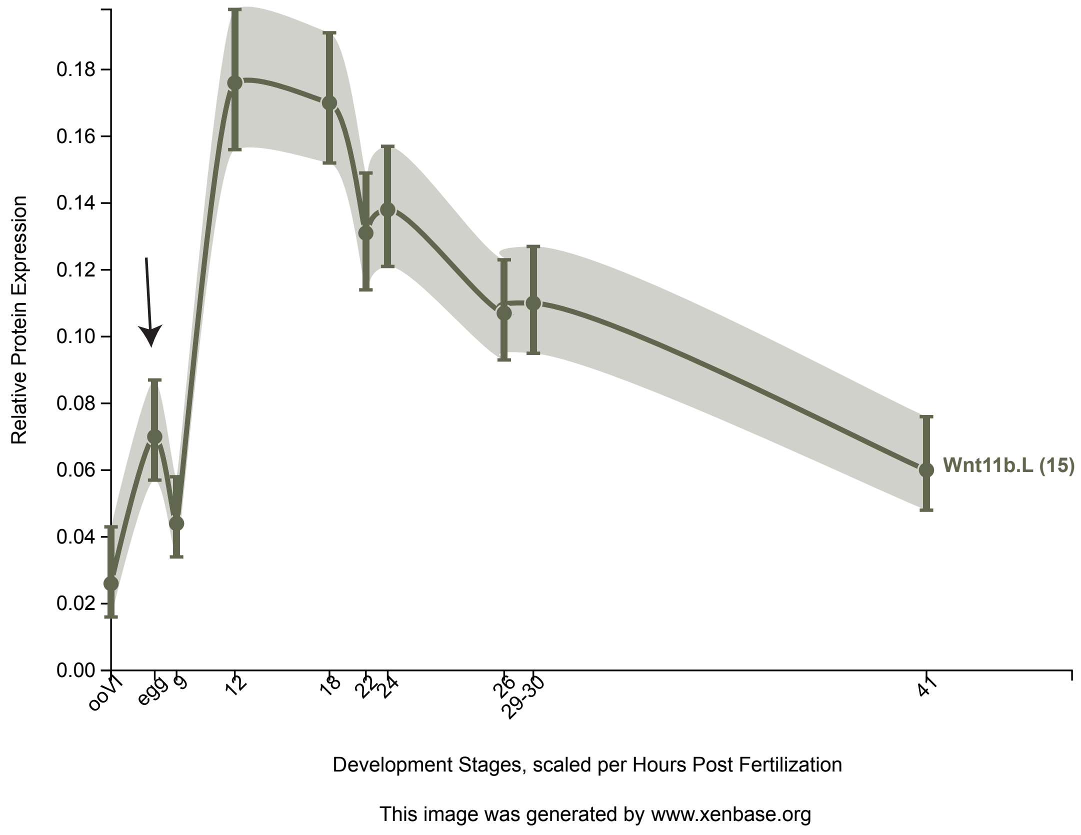

Fig. S10. Relative protein expression of Wnt11b protein during *Xenopus laevis* development. Data were visualized on xenbase.org and were derived from Peshkin et al. (2019). Nieuwkoop and Faber stages are shown on the x-axis, scaled to represent hours post-fertilization. The arrow indicates an increase in relative Wnt11b protein level in the fertilized egg. A larger increase is apparent during gastrulation.

Table S1. Primers and Oligos

wnt11b.L **CRISPR Guide RNAs**

Name	*CRISPRscan Score**	*Position*	*Exon*	*Length (bp)*	*Target Sequence (PAM)*
GG18NGG-72 (sgT1)	72	chr8L:21220561..21220583 (+ strand)	1	23	GGATCTGTGGCGCCATCCAG(TGG)
GG18NGG-44 (sgT2)	44	chr8L:21221317..21221339 (+ strand)	2	23	GGCTCACCGTAAATGGCAGC(CGG)

from: http://www.xenbase.org/common/displayJBrowse.do?data=data/xl9_2&loc=chr8L:21220220..21226166&tracks=XL9_2_Xenbase

*(Moreno-Mateos et al., 2015)

	Oligo Sequence
GG18NGG-72 (sgT1)	taatacgactcactataGGATCTGTGGCGCCATCCAGgttttagagctagaa
GG18NGG-44 (sgT2)	taatacgactcactataGGCTCACCGTAAATGGCAGCgttttagagctagaa
Universal Reverse Oligo	aaaagcaccgactcggtgccactttttcaagttgataacggactagccttattttaacttgctatttctagctctaaaac

wnt11b.L **Genotyping Primers**

Sequence	*Amplicon*	*Notes*
F: AAGCCACTGACTTGACCGC	-----	N/A
R: GCAGCTCTCATTGTGGTTTAG	703 bp	use for PCR product sequencing

wnt11b.L **CDS Primers**

Sequence	*Amplicon*	*Notes*
F: ATGGCTCCGACCCGTCAC	-----	N/A
R: CTTGCAGACATACCTCTC	1041 bp	no stop codon

RT-PCR Primers

Name	*Detection*	*Forward*	*Reverse*	*Reference*
odc1.L/S	UPL #69	F: CAGCTTCAGCAATGACGACT	R: AGATCAGCAACATAAAAGGCATC	Schneider et al., 2010
fgfr1.L/S	UPL #89	F: TCGGACAAGGATATGGAGGT	R: AATGGAGTTAGCGGCCAAG	prev. unpublished
wnt11b.L	UPL #16	F: CAGGATCTTCCCCATATTGC	R: CAGGATCTTCCCCATATTGC	prev. unpublished
wnt11.L/S	UPL #94	F: CGAATAAGCTCATGCACCTG	R: GAGACGCCATGACACTTGC	prev. unpublished
odc.L/S	SYBR	F: CATGCACATGTCAAGCCAGT	R: CAGGGAGAATGCCATGTTCT	prev. unpublished
fgfr1.L/S	SYBR	F: TCGCCCCTAAAACCAAAACG	R: TGTCATCATCATCATCGTCGTCC	Monsoro-Burq et al., 2003
myod1.L/S	SYBR	F: AGCTCCAACTGCTCCGACGGCATGAA	R: AGGAGAGAATCCAGTTGATGGAAACA	Rupp and Weintraub, 1991
sizzled.L.S	SYBR	F: GTCTTCCTGCTCCTCTGC	R: AACAGGGAGGACAGGAAG	prev. unpublished
sia1.L/S	SYBR	F: GAGCCCAGGATACAGGTTTG	R: CAGTTTGGGTAGGGCTGTGT	prev. unpublished
nodal3.1.L	SYBR	F: TAATCTGTTGTGCCGATCCA	R: ATCAATGTTGCCCTTTTTCA	Kofron et al., 2004

** primers and oligos are shown in 5' -> 3' orientation
UPL = Universal Probe Library hydrolysis probes

Development • Supplementary information

Table S2. Summary of microtubule defects in *wnt11b.L* mutant eggs

Series 1

female x male geno.	*Normal (1)*	*Reduced/Disorg. (2)*	*Severely reduced (3)*	*n*
60 minutes post-fertilization				
-/- x -/-	0	8	4	12
-/- x +/+	0	6	6	12
+/+ x -/-	12	0	0	12
+/+ x +/+	10	1	0	11
80 minutes post-fertilization				
-/- x -/-	0	2	10	12
-/- x +/+	0	5	7	12
+/+ x -/-	11	1	0	12
+/+ x +/+	8	2	0	10

**Series 2**	*Normal (1)*	*Reduced/Disorg. (2)*	*Severely reduced (3)*	*n*
-/- 70'; post-prick activation	7	12	1	20
+/+ 70' post-prick activation	18	2	0	20

Table S3. Summary of microtubule dynamic parameters in *wnt11b.L* mutant oocytes and eggs

**Oocytes (n=8 each)**	*wildtype_oocyte*	*mutant_oocyte*	*adj_p*	*sig (adj_p<0.01)*
nGrowths	1716.88 ± 507.16	2207.75 ± 1327.80	0.3452	FALSE
growth_speed_mean (µm/min)	6.96 ± 0.417	7.65 ± 0.38	0.0185	FALSE
growth_lifetime_mean (sec)	5.68 ± 0.22	5.43 ± 0.53	0.2943	FALSE
growth_length_mean (µm)	0.64 ± 0.051	0.69 ± 0.05	0.2533	FALSE
Rvalue	0.027 ± 0.019	0.041 ± 0.02	0.2943	FALSE

**Eggs (n=8 each)**	*wildtype_egg*	*mutant_egg*	*adj_p*	*sig (adj_p<0.01)*
nGrowths	1196.57 ± 384.14	1064.75 ± 345.60	0.6204	FALSE
growth_speed_mean (µm/min)	6.52 ± 0.47	7.29 ± 0.67	0.0632	FALSE
growth_lifetime_mean (sec)	6.69 ± 0.29	6.20 ± 0.25	0.0175	FALSE
growth_length_mean (µm)	0.68 ± 0.05	0.72 ± 0.05	0.2813	FALSE
Rvalue	0.06 ± 0.04	0.05 ± 0.05	0.6703	FALSE

**Activated eggs (n=12, 14)**	*wildtype_60min*	*mutant_60min*	*adj_p*	*sig (adj_p<0.01)*	*cvar_wildtype*	*cvar_mutant*
nGrowths	5197 ± 1159.96	3728.93 ± 1378.33	0.0310	FALSE	0.2232	0.3427
growth_speed_mean (µm/min)	8.25 ± 0.46	7.90 ± 0.77	0.5158	FALSE	0.0558	0.0975
growth_lifetime_mean (sec)	6.86 ± 0.42	6.39 ± 0.58	0.0879	FALSE	0.0612	0.0906
growth_length_mean (µm)	0.94 ± 0.07	0.81 ± 0.07	0.0015	**TRUE**	0.0745	0.0864
Rvalue	0.29 ± 0.07	0.16 ± 0.08	0.0028	**TRUE**	0.2414	0.5000

cvar= coefficient of variation

Development • Supplementary information

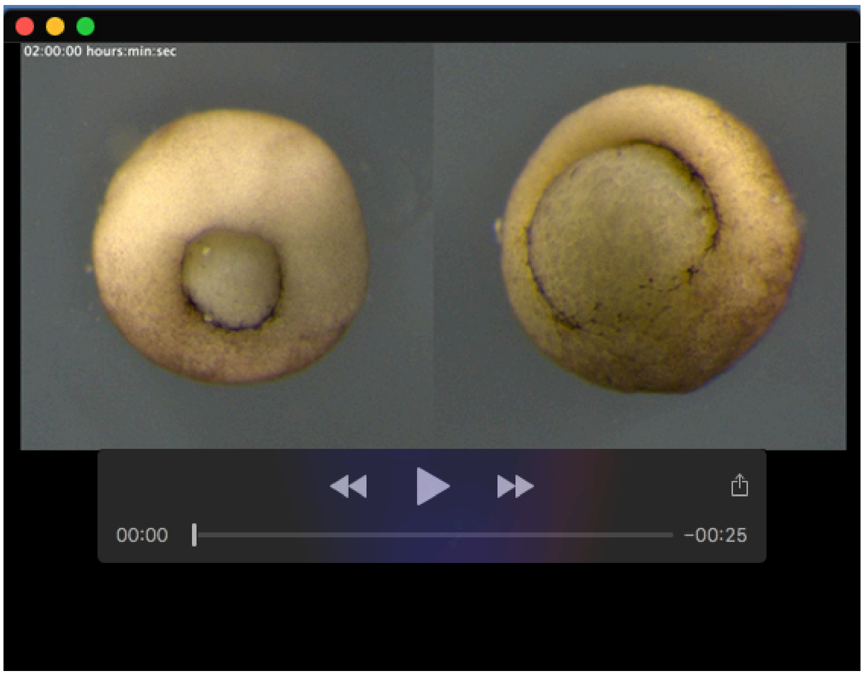

Movie 1. Gastrulation delay in *wnt11b* mutants. Representative time lapse movies of control heterozygous embryos (left) or *wnt11b-/-* mutant embryos (right). Embryos were imaged beginning around stage 10.5 and frames were captured every 10 minutes for nine hours. The time is displayed in the upper left; hr:min:sec. Related to Fig. 1E.

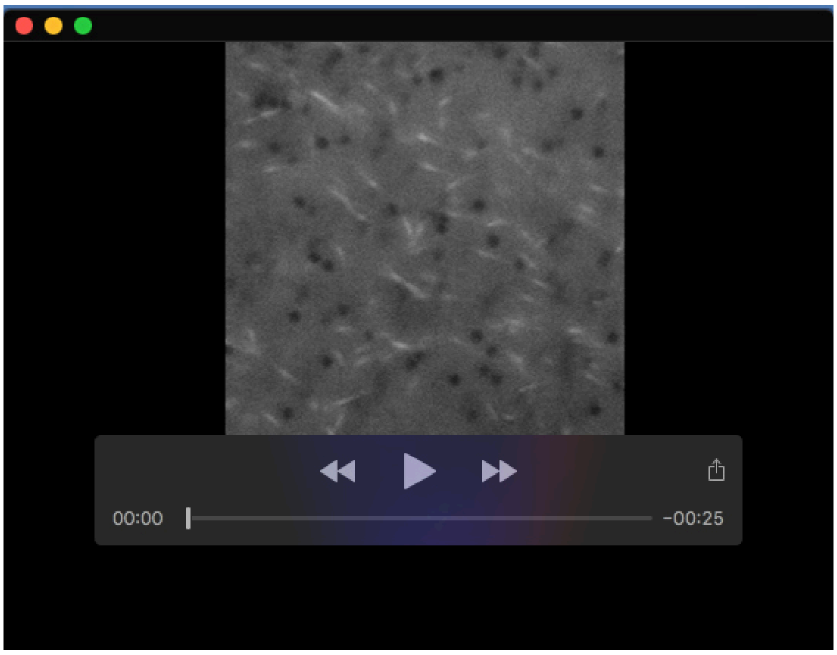

Movie 2. Representative time-lapse imaging of a prick-activated wildtype control egg injected with *eb3-gfp* mRNA (500 pg). Frames were captured at two-second intervals over two minutes. This movie represents normal cortical rotation; related to Fig. 7.

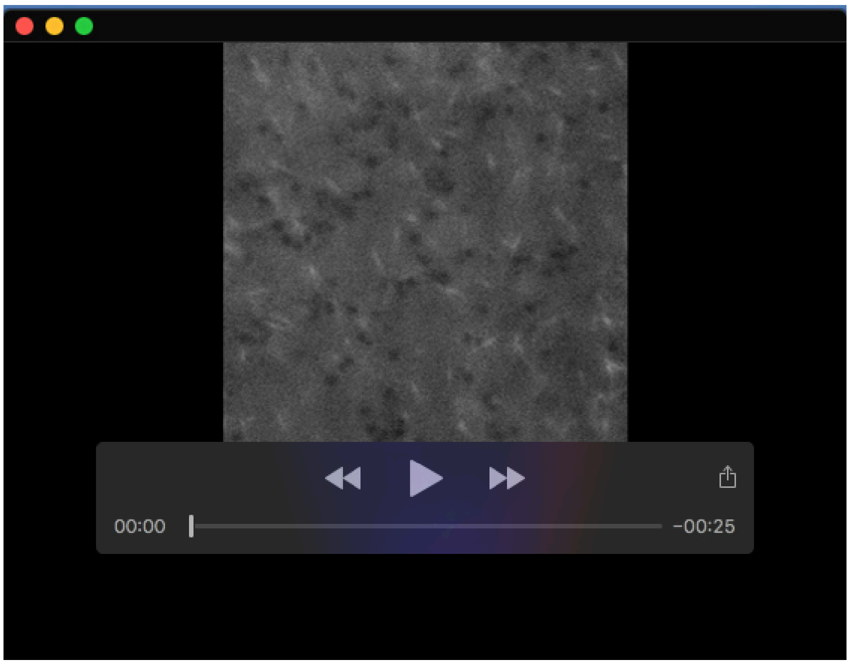

Movie 3. Representative time-lapse imaging of a prick-activated *wnt11b* -/- mutant egg injected with *eb3-gfp* mRNA (500 pg). Frames were captured at two-second intervals over two minutes. This movie represents reduced but directional cortical rotation; related to Fig. 7.

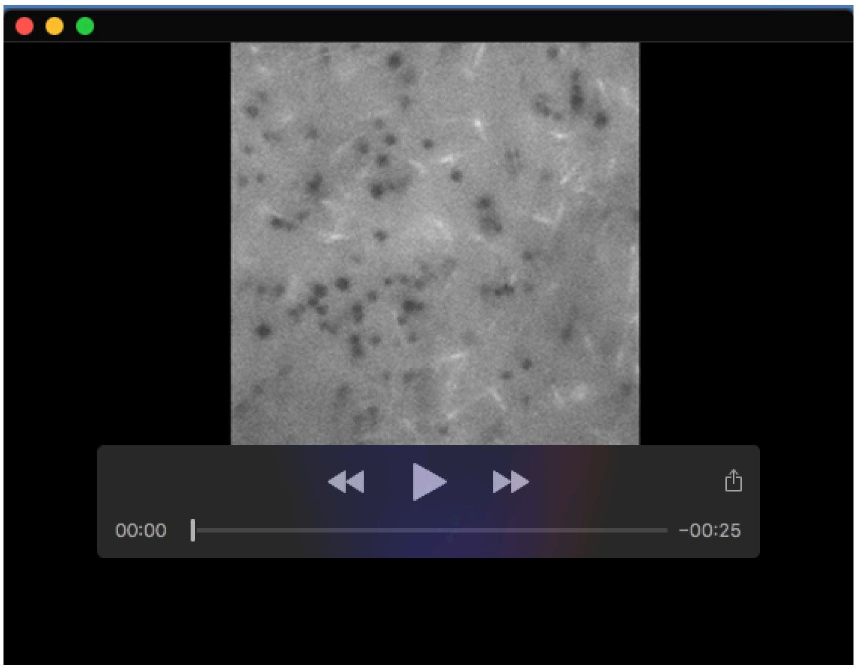

Movie 4. Representative time-lapse imaging of a prick-activated *wnt11b* -/- mutant egg injected with *eb3-gfp* mRNA (500 pg). Frames were captured at two-second intervals over two minutes. This movie represents reduced but poorly directional cortical rotation; related to Fig. S8.
