# Supplementary material for: Maternal Wnt11b regulates cortical rotation during Xenopus axis formation: analysis of maternal-effect wnt11b mutants
Source: Development. 2022 Sep 9;149(17):dev200552. doi: 10.1242/dev.200552 (PMC9515810; doi:10.1242/dev.200552)
Supplement: Reviewer comments [file develop-149-200552_review_history.pdf]

Development

Maternal Wnt11b regulates cortical rotation during *Xenopus* axis formation: analysis of maternal-effect *wnt11b* mutants

Douglas W. Houston, Karen L. Elliott, Kelsey Coppenrath, Marcin Wlizla and Marko E. Horb
DOI: 10.1242/dev.200552

Editor: Liz Robertson

Review timeline

Original submission:	3 February 2022
Editorial decision:	21 March 2022
First revision received:	3 July 2022
Editorial decision:	25 July 2022
Second revision received:	26 July 2022
Accepted:	1 August 2022

Original submission

First decision letter

MS ID#: DEVELOP/2022/200552

MS TITLE: Maternal Wnt11b regulates cortical rotation during *Xenopus* axis formation: analysis of maternal-effect *wnt11b* mutants

AUTHORS: Douglas W. Houston, Karen L Elliott, Kelsey Coppenrath, Marcin Wlizla, and Marko E. Horb

I have now received all the referees' reports on the above manuscript, and have reached a decision. The referees' comments are appended below, or you can access them online: please go to BenchPress and click on the 'Manuscripts with Decisions' queue in the Author Area.

As you will see, the referees express considerable interest in your work, but have some significant criticisms and recommend a substantial revision of your manuscript before we can consider publication. If you are able to revise the manuscript along the lines suggested, which may involve further experiments, I will be happy receive a revised version of the manuscript. Your revised paper will be re-reviewed by one or more of the original referees, and acceptance of your manuscript will depend on your addressing satisfactorily the reviewers' major concerns. Please also note that Development will normally permit only one round of major revision. If it would be helpful, you are welcome to contact us to discuss your revision in greater detail. Please send us a point-by-point response indicating your plans for addressing the referee's comments, and we will look over this and provide further guidance.

Please attend to all of the reviewers' comments and ensure that you clearly highlight all changes made in the revised manuscript. Please avoid using 'Tracked changes' in Word files as these are lost in PDF conversion. I should be grateful if you would also provide a point-by-point response detailing how you have dealt with the points raised by the reviewers in the 'Response to Reviewers' box. If you do not agree with any of their criticisms or suggestions please explain clearly why this is so.

© 2022. Published by The Company of Biologists under the terms of the Creative Commons Attribution License (https://creativecommons.org/licenses/by/4.0/).

Reviewer 1

Advance summary and potential significance to field

The authors have used CRISPR-mediated mutagenesis to create a loss of function allele in Wnt11b in Xenopus laevis. They have used this mutant to study maternal function of Wnt11b and observe clear and dramatic defects on developmental timing and gastrulation, as well as disruption of the parallel microtubule array in the egg.

Doing mutagenesis in X. laevis is heroic given the generation time (note the times from F0 to F2). The results are interesting and important for the field with the major and surprising conclusion that maternal Wnt11 is important in organizing the parallel microtubule array and that loss of Wnt11 has profound effects on developmental timing and cell movements at the gastrula stage. My comments address mostly minor changes to the text and figure legends.

Comments for the author

Suggested changes to the text and figure legends. No new experiments suggested.

1. Please explain the symbols in Figure 1A.
2. The data in Tables S2 and S3 providing the frequencies of phenotypes are important and could be moved to the main section of the manuscript, either combined with the relevant figures or as free standing tables. However, could they please provide more explanation for table S2? What does “partially ventralized” mean? What does “Open Bl./ect. Tailbuds” mean? I can guess but why not explain? Furthermore, “Ventralized” is an ambiguous and often misused term; it would be better to define more specifically which features they observe.
3. Are the results in Figure 4R and 4S statistically significant?
4. Is there developmental delay before gastrulation? For example, are cell cycle lengths similar, do Wnt11b mutants reach MBT at the same time as wt, do they initiate gastrulation at the same time? This is not a criticism or a suggestion for additional experiments; just an area that could be expanded on in the text if they have the data.
5. The rescue with injected Wnt11b RNA is important, but it was difficult to get a clear idea of who well the rescue worked. Also, the legend to Figure S5 is confusing; is there a typo? I find it difficult to understand which embryos are mutant and which were injected with RNA. Also, it would be very helpful to state the numbers of embryos injected, rescued, etc.
6. I do not understand the point of Figure 5, which shows that a phosphorylation event induced by progesterone is not dependent on Wnt11. What does this have to do with Wnt11 signaling? Additionally, phosphorylation at this site is not detectable in the presence of wild-type Wnt11, so why would one expect an effect in the mutant?
7. In Figure 7G, why are the x-axes on different scales for control vs mutant? To compare wt and mutant, shouldn’t the data be displayed on the same scale for each feature they measure? It seems that this might also show more clearly the wider variability in microtubule growth parameters for mutants vs wt.

Reviewer 2

Advance summary and potential significance to field

Wnt signalling is well known to play a key role in dorsal axis formation across vertebrates and Wnt11 has long been thought to act as a dorsal determinant in Xenopus. However, the differneces in phenotypes between knock-down in Xenopus versus mutants in zebrafish combined with paralogy redundancy, have confounded understanding of the precise roles of Wnt11. Houston and coworkers now report a Xenopus CRIPSR/Cas mutant affecting Xenopus Wnt11b and report that maternal Wnt11b plays key roles in axis formation and gastrulation, but not as a dorsal determinant itself, but rather, by regulation of cortical rotation. This is an unexpected and surprising finding. Moreover, the Wnt coreceptor Lrp6 and Wnt effector Dishevelled do not manifest roles consistent with beta-catenin stabilisation. Houston et al propose that Wnt11 functions by signalling to the cytoskeleton to enable robust cortical rotation. This is a novel hypothesis about Wnt11 function in early vertebrate axis formation.

© 2022. Published by The Company of Biologists under the terms of the Creative Commons Attribution License (https://creativecommons.org/licenses/by/4.0/).

Comments for the author

The work is of interest to developmental biologists, but the findings need to be strengthened. Some of the data is not presented well, some figure panels are mislabeled and the analysis can be quantitative and extended to better support the main claims.
1. In the maternal Wntt1b mutant embryos that fail to close or are delayed in closure of the blastopore, is the cytoskeleton disorganised? Is the gastrulation defect also owing to Wnt11b regulation of the cytoskeleton (in addition to its effects on cortical rotation)? Cytoskeletal organisation should be examined in the mutant versus wild type embryos.
2. With regards to the effect of maternal Wnt11b on cortical rotation, the authors show mutant and wild type embryos only at one time point (60 minutes). How many mutant embryos show disorganised microtubules?
In the supplemental table, the authors report that some mutant embryos (upto 20%) go on to form a normal axis and a further 30-35% only show partial ventralisation. Do the arrays not form at all in the mutants or could it be that microtubule arrays do form in these embryos, but perhaps slower than wild type or to varying extents? The authors should show microtubule organisation over a time series from fertilisation onwards (e.g. at 10 minute intervals for 90 minutes) in the mutants versus wild type embryos, and microtubule organisation should be analysed quantitatively.
3. In Figure 6, the authors show localisation of Dvl2-mCherry but the results describe distribution of Dvl2-GFP puncta. This should be corrected.
4. In figure 3, pitx2c expression in M/Z Wnt11b mutant embryos is only shown on the left and expression on the right side is not shown. This should be shown.
5,. In Fig 5, why is Lrp6 expression (detected by anti-Lrp6 cocktail) reduced in wild type embryos upon progesterone treatment(Fig 5A, B)? The levels of Lrp6 and phopho-Lrp6 should be quantitated in wild type versus mutant embryos.
6. Similarly, levels of Dvl2-GFP should be quantitated in wild type versus. mutant embryos (Fig 6 G).

First revision

Author response to reviewers' comments

Dear Dr. Robertson,

We are submitting our revised manuscript "*Maternal Wnt11b regulates cortical rotation during Xenopus axis formation: analysis of maternal-effect wnt11b mutants*" The reviewers have made valuable suggestions for improving the work and we thank them for their critiques. We have tried to address all the listed concerns, in many cases clarifying what we did not make clear in the first submission and adding small experiments in others. Below are our individual responses to the reviewers' points. The edited text is in blue in the revised manuscript; the responses to the numbered comments (bolded) below are indicated with a ">".

We hope these additions and changes are sufficient and we look forward to publishing this work in *Development*.

Responses:

Reviewer 1 Comments for the Author:
Suggested changes to the text and figure legends. No new experiments suggested.

1. ***Please explain the symbols in Figure 1A.***

> This explanation has been added to the **Figure 1** legend: "Females are represented by circles, males are represented by squares. The symbol in light grey indicates F0 mosaic; black indicates mutant at sgT1 site; dark grey indicates mutant at sgT2 site; full shading indicates homozygosity; half shading indicates heterozygosity. "

© 2022. Published by The Company of Biologists under the terms of the Creative Commons Attribution License (https://creativecommons.org/licenses/by/4.0/).

2. The data in Tables S2 and S3 providing the frequencies of phenotypes are important and could be moved to the main section of the manuscript, either combined with the relevant figures or as free standing tables. However, could they please provide more explanation for table S2? What does "partially ventralized" mean? What does "Open Bl./ect. Tailbuds" mean? I can guess but why not explain? Furthermore, "Ventralized" is an ambiguous and often misused term; it would be better to define more specifically which features they observe.

> We have added the relevant tables to the main manuscript; these are now **Tables 1 and 2**. Table legends have been added and the descriptions have been clarified. The "partially ventralized" terminology was revised to "DAI < 4", after the Kao and Elinson (1988) table.

3. Are the results in Figure 4R and 4S statistically significant?

> Those results were from single biological replicates, but we have since added a second biological replicate and calculated p values for these and the other PCRs. Other corrections were made; M/+ and M/Z samples were in the wrong order in **Fig. 4Q** and the error bars were actually standard deviations (not SEM). The figure legend was corrected. These did not change the findings of the experiments.

4. Is there developmental delay before gastrulation? For example, are cell cycle lengths similar, do Wnt11b mutants reach MBT at the same time as wt, do they initiate gastrulation at the same time? This is not a criticism or a suggestion for additional experiments; just an area that could be expanded on in the text if they have the data.

> We have not noted any differences prior to gastrulation, and we have not measured cell cycle lengths. However, dorsal lip formation appears to occur normally in the *wnt11b* mutants (or appears to at least be initiated). Also, we noted from the RNA seq data that *gs17.L* (a marker used for MBT onset) expression was similar in mutants and heterozygotes, further arguing against a developmental delay at this level. Additional text has been added on p.5 and examples of early gastrula in **Supp. Fig. S2A-C**. Gs17 was included in the heatmaps in **Fig. S4** and its expression mentioned on p. 8.

5. The rescue with injected Wnt11b RNA is important, but it was difficult to get a clear idea of how well the rescue worked. Also, the legend to Figure S5 is confusing; is there a typo? I find it difficult to understand which embryos are mutant and which were injected with RNA. Also, it would be very helpful to state the numbers of embryos injected, rescued, etc.

> The figure legend text had incorrectly referred to "Samples in A were left uninjected .." (A is the PCR data); this has been corrected. Also, annotation was added to the figure panels to indicate genotype and +/- mRNA injection. The numbers were presented in **Table S2**, which has been moved into the main text (now Table 1). Numbers from a subsequent experiment have been added.

6. I do not understand the point of Figure 5, which shows that a phosphorylation event induced by progesterone is not dependent on Wnt11. What does this have to do with Wnt11 signaling? Additionally, phosphorylation at this site is not detectable in the presence of wild-type Wnt11, so why would one expect an effect in the mutant?

> This experiment was originally conceived to test whether Lrp6 was phosphorylated in the *oocyte*, and whether this was regulated by Wnt11, following up Kofron et al 2007. Because Wnt11 translation seems to be stimulated during oocyte maturation (i.e. after progesterone treatment) (Van Itallie et al., 2022; Peshkin et al., 2019 - visualised on Xenbase) it was possible that Lrp6 phosphorylation could be dependent on Wnt11 signalling in the *egg*. Panel C of **Fig. 5** shows that neither the phosphorylation of Lrp6 nor its dephosphorylation require Wnt11b.

7. In Figure 7G, why are the x-axes on different scales for control vs mutant? To compare wt and mutant, shouldn't the data be displayed on the same scale for each feature they measure? It seems that this might also show more clearly the wider variability in microtubule growth parameters for mutants vs wt.

© 2022. Published by The Company of Biologists under the terms of the Creative Commons Attribution License (https://creativecommons.org/licenses/by/4.0/).

> In this case, those correlation plots were made with MATLAB's 'corrplot' command, which plots correlations for all pairs of variables in the dataset, and the internal software scales the axes by default to the min/max of each variable in that dataset. Since the range of values for wildtype and mutant samples were different, the axes ended up different. However, it is indeed better to have consistent axes, so we have replotted the panels with consistent axis values (**Figure 7G**).

Reviewer 2 Comments for the Author:
The work is of interest to developmental biologists, but the findings need to be strengthened. Some of the data is not presented well, some figure panels are mislabeled and the analysis can be quantitative and extended to better support the main claims.

1. *In the maternal Wnt11b mutant embryos that fail to close or are delayed in closure of the blastopore, is the cytoskeleton disorganised? Is the gastrulation defect also owing to Wnt11b regulation of the cytoskeleton (in addition to its effects on cortical rotation)? Cytoskeletal organisation should be examined in the mutant versus wild type embryos.*

> This is an important but very broad question, and one that would be difficult to fully address in the context of this work. We did examine gross cytoskeletal organization using probes/mAbs against actin, tubulin and cytokeratin and found no obvious differences, although there were hints that microtubule and cytokeratin organziation might be *more* extensive, which could implicate Wnt11b signaling in reducing or promoting organizaiton of these cytoskeletal systems. A rigorous quantitative analysis of all the dynamics of these molecules would be highly interesting and we hope that our reporting of the phenotype and our crude results in this aspect of Wnt11 function will interest others in picking up on this line of research. We have inserted these data along with the gastrula images from Reviewer 1's point #4 in **Fig. S2** and added some text to clarify (p. 6).

2. With regards to the effect of maternal Wnt11b on cortical rotation, the authors show mutant and wild type embryos only at one time point (60 minutes). How many mutant embryos show disorganised microtubules?

In the supplemental table, the authors report that some mutant embryos (up to 20%) go on to form a normal axis and a further 30-35% only show partial ventralisation. Do the arrays not form at all in the mutants or could it be that microtubule arrays do form in these embryos, but perhaps slower than wild type or to varying extents? The authors should show microtubule organisation over a time series from fertilisation onwards (e.g. at 10 minute intervals for 90 minutes) in the mutants versus wild type embryos, and microtubule organisation should be analysed quantitatively.

> Unfortunately the numerical data and the fact that we examined two time points was not made clear in the relevant section (p,10-11). Microtubule organisation was similarly disrupted at 60' and 80' post-fertilization (and at 70' post-activation). We have added clarifying text to that section (p. 11) and included numbers w/ percentages in addition to referring to the Supplemental Table (now **Table S2**).

Also, we performed the suggested time course analysis using plus end imaging with a small number of samples (n=3); the wildtype eggs showed the expected increase in average directionality (the "resultant vector magnitude" r) whereas the mutant eggs showed no significant changes. (Presented in **Fig. 7E-F**, p. 12). There was a statistically insignificant/slight increase in r at the 85' mutant time point, but this doesn't represent significant alignment and is near the end of cortical rotation (which is terminated by first cleavage at 90') in any case, and thus likely not responsible for "rescuing" axis defects. A more probable explanation for normal axial development in the mutants is the inherent stochasticity of cortical rotation in general; Vincent and Gerhart (1987) showed that normal embryos can result from a wide range of cortical displacement (from 13-35° of arc, which can overlap with ranges in UV ventralized embryos). Additional discussion was added on p.16.

The analysis by plus end tracking that we have used is most reliable way to objectively assess microtubule organisation (at least for parallel alignment) as this method is a reliable proxy both for direction (r) and extent (numbers of growing plus ends) and there does not appear to be generally accepted tool for measuring these from static images (although quite easy to see qualitatively).

© 2022. Published by The Company of Biologists under the terms of the Creative Commons Attribution License (https://creativecommons.org/licenses/by/4.0/).

3. In Figure 6, the authors show localisation of Dvl2-mCherry but the results describe distribution of Dvl2-GFP puncta. This should be corrected.

> We were unclear in that section; **Fig. 6** panels (A-F) and (G) are indeed Dvl2-GFP; (H-I) are Dvl2-mCherry with Rab-GFPs. We added a label on the figure to indicate that Dvl2-GFP is being expressed in those panels and clarified the text (p.10).

4. In figure 3, pitx2c expression in M/Z Wnt11b mutant embryos is only shown on the left and expression on the right side is not shown. This should be shown.

> The panel showing the right sides of M/Z Wnt11b embryos (lacking expression and with bilateral expression) has been added to **Fig. 3**. This figure panel has also been reconfigured for better comparison of the data.

5,. In Fig 5, why is Lrp6 expression (detected by anti-Lrp6 cocktail) reduced in wild type embryos upon progesterone treatment(Fig 5A, B)? The levels of Lrp6 and phospho-Lrp6 should be quantitated in wild type versus mutant embryos.

> It seems likely that the S1490 phosphorylation partially blocks the epitope for the non-phospo antibodies (although this epitope is either not known or provided by the antibody provider). Note that the band is shifted in the progesterone-treated samples, but the intensity is lower, suggesting that phosphorylation is occurring but not not as well detected. Because of this likelihood, we only quantified wildtype versus mutant as pairs for the phospho Lrp6 samples (it probably would not be meaningful to compare total Lrp6 in the presence of the potentially blocking phosphorylation). These quantification data were added below the panel.

6. Similarly, levels of Dvl2-GFP should be quantitated in wild type versus. mutant embryos (Fig 6 G).

> This was repeated (using oocytes from a different female) and the averaged values for the quantified bands shown in a box and whisker chart in a new panel (**Fig. 6 G'**). None of the differences are significant (by anova).

Second decision letter

MS ID#: DEVELOP/2022/200552

MS TITLE: Maternal Wnt11b regulates cortical rotation during *Xenopus* axis formation: analysis of maternal-effect *wnt11b* mutants

AUTHORS: Douglas W. Houston, Karen L Elliott, Kelsey Coppenrath, Marcin Wlizla, and Marko E. Horb

I have now received all the referees reports on the above manuscript, and have reached a decision. The referees' comments are appended below, or you can access them online: please go to BenchPress and click on the 'Manuscripts with Decisions' queue in the Author Area.

The overall evaluation is very positive and we would like to publish a revised manuscript in Development. However as you'll see Reviewer 2 has a very minor suggestion to modify the Discussion that I hope you can address in the very final version of your manuscript. Please attend to all of their comments in your revised manuscript and detail them in your point-by-point response. If you do not agree with any of their criticisms or suggestions explain clearly why this is so. Your paper will not require any further re-review, rather I will look it over myself prior to acceptance.

© 2022. Published by The Company of Biologists under the terms of the Creative Commons Attribution License (https://creativecommons.org/licenses/by/4.0/).

Reviewer 1

Advance summary and potential significance to field

A CRISPR-generated loss of function mutation in Xenopus laevis Wnt11b causes dramatic defects in the parallel microtubule array, developmental timing, and gastrulation. Mutagenesis in X. laevis is heroic given the generation time and complexity of the duplicated genome. The results are interesting and important for the field, providing both conceptual and technological advances.

Comments for the author

The authors fully addressed my comments on the first review.

Reviewer 2

Advance summary and potential significance to field

The revised m/s has improved and many of the concerns have been addressed. I would like some fo the explanation provided in the response to reviews to be included in the discussion. Specifically, "there were hints that microtubule and cytokeratin organziation might be more extensive, which could implicate Wnt11b signaling in reducing or promoting organizaiton of these cytoskeletal systems." should be added to the discussion.

Comments for the author

The revised manuscript has improved and most of the concerns have been addressed.

One minor suggestion is to include the response to the comment regarding the effect on the cytoskeleton to the discussion: "there were hints that microtubule and cytokeratin organization might be more extensive, which could implicate Wnt11b signaling in reducing or promoting organizaiton of these cytoskeletal systems."

Second revision

Author response to reviewers' comments

Dear Dr. Robertson,

We are submitting our revised manuscript "*Maternal Wnt11b regulates cortical rotation during Xenopus axis formation: analysis of maternal-effect wnt11b mutants*" The reviewers have made valuable suggestions for improving the work and we thank them for their critiques. We made a few typo/corrections, and the changes from the previous submission have been reverted to black text. Only the revised section recommended by Reviewer 2 is in blue text (also below).

We hope these additions and changes are sufficient and we look forward to publishing this work in *Development.*

Responses:

Reviewer 1 Comments for the Author:
The authors fully addressed my comments on the first review.

> N/A

© 2022. Published by The Company of Biologists under the terms of the Creative Commons Attribution License (https://creativecommons.org/licenses/by/4.0/).

Reviewer 2 Comments for the Author:
The revised manuscript has improved and most of the concerns have been addressed.
One minor suggestion is to include the response to the comment regarding the effect on the cytoskeleton to the discussion: "there were hints that microtubule and cytokeratin organization might be more extensive, which could implicate Wnt11b signaling in reducing or promoting organizaiton of these cytoskeletal systems."

> This is a good suggestion. We have added an edited version of the above sentence to the end of paragraph 2 on page 16 in the discussion and further revised an unfortunate final sentence of that paragraph. The original text:

“A preliminary analysis of cytoskeletal organisation failed to identify substantial differences in mutant gastrulae, but a more thorough analysis and quantitative analysis of overall cytoskeletal analysis is warranted.”

Has been changed to (underlined are revisions for clarity and to better reflect original intent):

“A preliminary analysis of cytoskeletal organisation failed to identify substantial differences in mutant gastrulae, but there were hints that microtubule and cytokeratin organisation might be more extensive, possibly implicating Wnt11b signaling in reducing or promoting reorganization of these cytoskeletal systems in different contexts. A more thorough and quantitative analysis of overall cytoskeletal dynamics in these embryos is warranted. “

Third decision letter

MS ID#: DEVELOP/2022/200552

MS TITLE: Maternal Wnt11b regulates cortical rotation during *Xenopus* axis formation: analysis of maternal-effect *wnt11b* mutants

AUTHORS: Douglas W. Houston, Karen L Elliott, Kelsey Coppenrath, Marcin Wlizla, and Marko E. Horb
ARTICLE TYPE: Research Article

I am happy to tell you that your manuscript has been accepted for publication in Development, pending our standard ethics checks.

© 2022. Published by The Company of Biologists under the terms of the Creative Commons Attribution License (https://creativecommons.org/licenses/by/4.0/).
